# Prediction of adolescent depression from prenatal and childhood data from ALSPAC using machine learning

**DOI:** 10.1038/s41598-024-72158-9

**Published:** 2024-10-07

**Authors:** Arielle Yoo, Fangzhou Li, Jason Youn, Joanna Guan, Amanda E. Guyer, Camelia E. Hostinar, Ilias Tagkopoulos

**Affiliations:** 1 Department of Computer Science, University of California – Davis, Davis, USA; 2 Genome Center, University of California – Davis, Davis, USA; 3 USDA/NSF AI Institute for Next Generation Food Systems (AIFS), Davis, USA; 4 Department of Psychology, University of California – Davis, Davis, USA; 5 Center for Mind and Brain, University of California – Davis, Davis, USA; 6 Department of Human Ecology, University of California – Davis, Davis, USA

**Keywords:** Psychology, Computer science

## Abstract

Depression is a major cause of disability and mortality for young people worldwide and is typically first diagnosed during adolescence. In this work, we present a machine learning framework to predict adolescent depression occurring between ages 12 and 18 years using environmental, biological, and lifestyle features of the child, mother, and partner from the child’s prenatal period to age 10 years using data from 8467 participants enrolled in the Avon Longitudinal Study of Parents and Children (ALSPAC). We trained and compared several cross-sectional and longitudinal machine learning techniques and found the resulting models predicted adolescent depression with recall (0.59 ± 0.20), specificity (0.61 ± 0.17), and accuracy (0.64 ± 0.13), using on average 39 out of the 885 total features (4.4%) included in the models. The leading informative features in our predictive models of adolescent depression were female sex, parental depression and anxiety, and exposure to stressful events or environments. This work demonstrates how using a broad array of evidence-driven predictors from early in life can inform the development of preventative decision support tools to assist in the early detection of risk for mental illness.

## Introduction

Depression is an impairing and prevalent disorder, affecting approximately 34% of adolescents aged 10–19 years globally^1^. Depression is also one of the leading causes of non-fatal disability^2^ and a major risk factor for suicide, the second leading cause of death for people aged 10–34 in the United States^3^. The main symptoms of depression include low mood, diminished interest or pleasure, change in sleep, weight or appetite, decrease in energy, feelings of worthlessness or guilt, and frequent thoughts of death or suicide^4^. Although these symptoms most often reach clinical levels of concern leading to diagnosis during adolescence, the developmental processes resulting in adolescent depression start years prior^5^, potentially even during gestation^6^. Through a hypothesized mechanism of stress sensitization, exposure to adverse early-life experiences such as parental mental illness or financial hardship is thought to increase the risk of future depression^7,8^. The connection between challenging childhood experiences and the risk of depression offers a chance to identify at-risk children early. Identification can be done by combining information about stressful events children have faced with data from comprehensive developmental assessments linked to depression risk (e.g., socio-emotional, cognitive, and biological). Early identification by age 10 of children at risk of developing depression during their adolescent years would provide new avenues for preemptive interventions, thereby reducing suffering, adolescent mortality, and treatment costs associated with depression^9,10^.

One prominent depression model is the multilevel biopsychosocial model put forth by Garber^5^. This model is based on evidence that depression is a complex condition influenced by various social-contextual, psychological, and biological factors, with no single factor accounting for a significant portion of the risk for depression^5^. Accordingly, individual factors (e.g., cognitive vulnerabilities, temperament, biological factors such as elevated inflammation or physical health problems) and social-contextual risk factors (e.g., stressful life events, poor quality of relationships with parents and peers, family socioeconomic status, neighborhood conditions) combine dynamically across development to heighten the risk for depression^5^. More recent evidence supports this model, illustrating the premise that no single risk factor causes depression and rather that multiple social, cognitive, and biological factors are linked to increased risk of depression^11–13^. Several of these factors or features have been identified as being particularly important for predicting depression. One of the most important features is parental depression, as parental history of major depression disorder (MDD) increases a child’s likelihood of being diagnosed with MDD three to fivefold^14–16^. Other important features include child gender (adolescent girls and women are at higher risk for depression)^17^, stressful family and social circumstances^18–20^, and cognitive and emotion regulation deficits^21,22^. Some of these features also relate to other features that influence mental health in general, such as parental incarceration affecting income and employment opportunities^23,24^, which makes this problem complex as non-linear interactions between multiple features can contribute to depression. Challenges encountered within previous studies aiming to predict adolescent depression include small sample sizes^25^ and the use of cross-sectional data that does not assess depression frequently^26^. Because depression is complex and multifaceted, predicting it accurately requires methods that can consider numerous factors and offer reliable measures for each potential outcome.

Machine Learning (ML) is a process where machines use algorithms to detect patterns from data without being explicitly programmed^27^. ML has led to unprecedented advances in many areas of medicine, from diagnosing heart disease^28^ and cancer^29^ to detecting schizophrenia through speech recognition^30^. ML is well-suited for learning from large multidimensional datasets in order to predict later clinical outcomes. More specifically, supervised ML methods use labeled data to make predictions on unseen data by minimizing the predictive error on the observed training data^31^ and could be useful for efforts to predict clinical outcomes such as depression. This process allows the method to learn the relations between the independent variables and the labeled dependent variable^31^. Some of the ML algorithms perform predictions by analyzing data cross-sectionally (i. e. without taking into account when each feature was recorded, thus making each observation a separate feature). In contrast, there are also time-series ML methods where data for the same construct recorded at different times is considered as the same feature containing a series of observations indexed by time^32^. Generally, cross-sectional data is much easier to obtain than time-series data thus making cross-sectional ML methods easier to apply. However, time-series ML methods are better for modeling temporal disease dynamics^33,34^. While there is some consensus on the kinds of ML algorithms that are appropriate for certain tasks, in general, it is difficult to determine a priori the algorithm that will perform best on a specific dataset^35^. Since prior work comparing models for depression prediction has not considered time-series model architectures^36,37^, it remains unknown whether models designed for time-series data could provide an advantage for depression prediction in comparison to simpler models that are more agnostic to temporality. Thus, we evaluated both approaches, one that considers the temporal sequence of the features in its architectural design and one that does not, as each approach could yield unique insights into the most predictive features.

Depression is typically diagnosed using clinical interviews and screening questionnaires with thresholds to determine whether the individual meets the criteria for a diagnosis of depression (e.g., the *Diagnostic and Statistical Manual of Mental Disorders* (DSM) criteria for MDD)^38^. For predicting depression, ML has been used in a sample of patients with brain injury^39^ in a large study of 6588 Korean adults^40^. ML has also been used to detect current depression based on the content of Twitter posts^41^, wearable mobile sensor data^42^, patterns of brain activity captured using electroencephalography data^43^, and to predict treatment outcomes for depression in adults^44–46^. For predicting adolescent mental health and depression trajectories years in advance, it was found that prediction accuracy results were mixed in terms of clinical relevance^47,48^. Additionally, prior research used ML methods for predicting depression applied to the Avon Longitudinal Study of Parents and Children (ALSPAC) dataset which includes demographic, clinical, and survey features^49,50^. This prior work focused on classifying depression trajectories from adolescence through adulthood^51^ and predicting depression during early adulthood (ages 23 through 28)^52^. Although features related to adult outcomes of depression are important to consider in depression development, we were interested in identifying a general model for predicting adolescent depression since the first onset of depression is often in adolescence^53^ and early intervention opportunities may improve adult outcomes^9,10^.

In this work, we built ML predictors for adolescent depression using the ALSPAC dataset, focusing on features from the prenatal period to age 10. To this end, we generated six different datasets to predict adolescent depression at five different time points (ages 12, 13, 16, 17, and 18 years) as well as to predict having depression at any time during these time points. We focused on these time points because the incidence of depression in the population increases steadily from age 12 to 18^54^, with many individuals having their first depression onset within this developmental period^53^. Through both statistical feature analysis and recursive feature elimination (RFE)^55^, we were able to identify and select the subset of features critical to the accurate prediction of adolescent depression and build predictors with an average recall of 0.59 and specificity of 0.61.

## Methods

### Dataset

The samples in this study consist of participants from the ALSPAC^49,50^. ALSPAC is an ongoing birth cohort study that has been following more than 14,000 participants from the prenatal period into adulthood to understand the role of environmental and genetic factors in shaping a wide range of developmental and health outcomes. Mothers were recruited if they had an expected delivery date between April 1, 1991, and December 31, 1992, and lived in the former county of Avon in the United Kingdom (UK). There were 20,248 eligible pregnancies in this region and time period. The initial recruitment resulted in a sample of 14,541 pregnant mothers, resulting in 14,676 fetuses, 14,062 of whom were alive at birth and 13,988 children who were alive at one year of age. Later efforts to bolster the initial sample with eligible cases who had failed to join the initial study yielded a total sample size for analyses of 15,645. A 10% sample of the ALSPAC cohort, known as the Children in Focus (CiF) group, attended clinics at the University of Bristol at various time intervals between 4 and 61 months of age. The CiF group was chosen at random from the last 6 months of ALSPAC births (1432 families attended at least one clinic). Excluded were those mothers who had moved out of the area or were lost to follow-up and those partaking in another study of infant development in Avon. The ALSPAC dataset includes many waves of data collection, including questionnaires completed by children, parents, and teachers; administrative records; observational data; clinical assessments; and biological samples. The study website contains details of all available data through a fully searchable data dictionary and variable search tool: http://www.bristol.ac.uk/alspac/researchers/our-data/. For further information regarding sample enrollment, participant characteristics, and general study methodology, refer to publications from the ALSPAC team that have profiled this cohort^49,50^. Ethical approval for the study was obtained from the ALSPAC Ethics and Law Committee and the Local Research Ethics Committees. Informed consent for the use of the data collected via questionnaires and clinics was obtained from participants following the recommendations of the ALSPAC Ethics and Law Committee at the time. All methods were performed in accordance with relevant guidelines and regulations, and all participants gave informed consent.

### Sample description

**Fig. 1:**
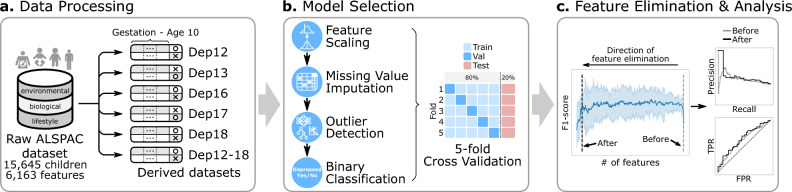
Overview of the adolescent depression prediction framework. (**a**) The Avon Longitudinal Study of Parents and Children (ALSPAC) dataset is a long-term study spanning over two decades since the early 90 s in the Bristol, UK area, which includes features like questionnaires, hospital records, and lab samples of the child, mother, and her partner from the gestation stage through adolescence. We use this ALSPAC dataset to generate 6 derivative datasets, five for predicting depression at each target age (12, 13, 16, 17, and 18) and one for predicting depression diagnosis any time between ages 12–18, using features from the gestation stage to age 10. These are represented in the figure as Dep12, Dep13, Dep16, Dep17, Dep18, and Dep12-18. (**b**) The model selection pipeline selects the best combination of feature selection (FS), missing value imputation (MVI), outlier detection (OD), and binary classification (CLS) for each derived dataset. For each combination, we also performed hyperparameter tuning using a fivefold cross-validation optimized for F1-score. (**c**) Once the best model pipeline is selected for each dataset, we run the recursive feature elimination (RFE) to reduce the number of features while retaining the model performance. TPR stands for true positive rate and FPR stands for false positive rate.

From the original ALSPAC dataset, we gained access to 6163 features from 15,645 participants measured at different time points and used data from 8467 participants with depression data at one or more time points from age 12 to age 18. Features represented major domains of child functioning (cognitive, social, emotional, and biological), as well as captured known risk factors for depression, based on prior theory^5,11,12^ (Fig. 1, Table 1). Some of these domains include socioeconomic factors, child-peer relationships, child psychopathology, child physical health, and parents’ perceived social support. See Table 1 for a list of all domains and ages recorded. We pruned the dataset by merging the features that are potential duplicates, relevant to only a specific subset of samples, and consistent with prior publications (Supplementary Information 1.1), resulting in 885 features.

**Table 1:** Domains and periods covered by the unique features used across the models.

Domains	Prenatal	Ages 0–2	Ages 3–5	Ages 6–7	Age 8	Age 9	Age 10
Child sex at birth		X					
Socioeconomic factors (parents, grandparents, neighborhood)*	X	X	X	X	X	X	X
Parental marital status (divorce, separation, etc.)	X	X	X	X	X	X	X
Parent–child relationship (e.g., observed interaction, self-reports)		X	X	X	X	X	
Child-peer relationships (e.g., friendships, popularity, prosociality, antisociality)				X	X	X	
Child temperament (e.g., activity, approach, adaptability, emotionality, distractibility, shyness)		X	X	X			
Child psychopathology (e.g., depression, emotional and behavioral difficulties)			X	X		X	X
Child self-esteem & locus of control					X	X	
Child biomarkers (e.g., CRP, IL6, parathyroid, cotinine, HDL, Vitamin D, calcium, albumin, hemoglobin, leptin)				X		X	
Child anthropometrics & physiology (BMI, fat mass assessed with DEXA, hip, waist, height, heart rate)				X	X	X	X
Child cognition, school-related variables, & extracurriculars				X	X	X	
Child physical health (e.g., general health, hospitalizations)		X	X	X	X		X
Child athletics/physical activity & physical appearance				X	X	X	
Child pubertal development					X	X	X
Child stressful life events, trauma, maltreatment (e.g., physical abuse, sexual abuse, separations from parents)		X	X	X	X	X	X
Parental psychopathology (e.g., depression) & stress	X	X	X	X	X	X	
Parents’ perceived social support and social network index	X	X	X	X		X	

*Socioeconomic factors included measures of educational attainment, income, social class score based on occupation (CAMSIS score) assessed at multiple ages, as well as the neighborhood quality index, neighborhood stress score, and mother’s and father’s score for their opinion of whether their neighborhood is a good place to live.

### Depression variable description

We used the Short Mood and Feelings Questionnaire (SMFQ)^56^ from the ALSPAC dataset as our depression variable. The SMFQ is a validated depression screening questionnaire^56–58^ that scores questions related to core depression symptoms between 0 and 26 using the interval scale^56^. We used the SMFQ to assign the dependent binary variable (depressed versus not depressed) at each target age (12, 13, 16, 17, and 18). Children with a score < 12 were assigned to the class ‘not depressed’ (label 0)’ and children with a score of 12 or higher were assigned to the class ‘depressed’ (label 1)’. Note that ALSPAC did not include the SMFQ measure for ages 11, 14, and 15, and thus, our analysis excludes the prediction at these ages. Features from the gestation period up to 10 years old (inclusive of age 10) were used as independent features.

### Cross-sectional data cleaning

We then processed this ALSPAC dataset in two different ways to be used for downstream ML analyses, resulting in two types of derivative datasets. The first dataset type was used to predict adolescent depression at each of the five target ages. To this end, for each target age, we cleaned the dataset by first removing the samples with a missing target variable, reducing the sample size (Supplementary Table 1, Supplementary Spreadsheet 1). We then removed the independent features that were obtained after ten years old, were constant, or had a proportion of missing values greater than or equal to 60% (Supplementary Figs. 1–7, Supplementary Table 2). We chose this missing value cutoff because it retained the most number of features without necessitating imputing a large majority of the data. Finally, we reduced the feature space by calculating the Pearson correlation coefficient between all pairwise combinations of the independent features and the target variable. We used a threshold of *p*-value < 0.05 to discard the independent features that were not significantly correlated with the target depression outcome variable (Supplementary Table 1, Supplementary Spreadsheet 1).

In this work, we refer to these cross-sectional datasets prepared for each target age as *Dep12* (6715 samples, 255 features), *Dep13* (6015 samples, 279 features), *Dep16* (4993 samples, 338 features), *Dep17* (4496 samples, 318 features), and *Dep18* (3334 samples, 299 features), respectively. We also created an additional type of dataset to predict adolescent depression anytime between ages 12 and 18. We were interested in predicting adolescent depression anytime between ages 12 and 18 in order to assess the general risk of a child becoming depressed during adolescence in addition to specific target ages. For this objective, we merged the dependent variables at five target ages into one by assigning ‘not depressed (label 0)’ if the child had a score below the clinical cutoff at all five time points and ‘depressed (label 1)’ if the child had a score that was above the clinical cutoff for at least one of the five time points. We refer to the resulting dataset that has 1799 samples and 266 features as *Dep12-18* in the manuscript. The number of samples and features after each data cleaning step can be found in Supplementary Table 1. Correlational statistics linking the predictors to depression at each time point and missing value ratios for the predictors can be found in Supplementary Spreadsheet 1.

### Cross-sectional model selection

To select the best model configuration that predicts the depression status of an adolescent, we designed an exhaustive model selection pipeline to select the optimal combination of preprocessing options and a classifier. The pipeline was developed primarily using the scikit-learn^60^, imbalanced-learn^61^, and MLxtend^62^ Python packages. The overall steps included preprocessing, which transformed the data such that it can be used for ML methods, and classification, which used the transformed data to learn relations among the predictors and depression. During the preprocessing step, the pipeline included, in the mentioned order, a categorical encoding method (one-hot encoding), three feature scaling (FS) methods (standard, minmax, robust^63^), three missing value imputation (MVI) methods (k-Nearest Neighbors (KNN)^64^, Multivariate Imputation by Chained Equations (MICE)^59^, MissForest^65^), and three outlier detection (OD) methods (isolation forest (IF)^66^, local outlier factor (LOF)^67^, no outlier removal (none)).

During the classification step, the pipeline included seven binary classification (CLS) methods (decision tree^68^, gaussian naïve Bayes (NB)^69^, multinomial NB^70^, support vector classifier (SVC)^71^, AdaBoost^72^, RandomForest^73^, multilayer perceptron (MLP)^74^) with the synthetic minority over-sampling technique (SMOTE)^75^ for up-sampling and a grid search to find the optimal hyperparameters. Up-sampling makes the dataset balanced while training so that the classifier is less biased toward predicting the majority class^75^, which in this case was “not depressed”. For each dataset, we randomly selected 20% for a held-out test set, and the rest was used for fivefold cross-validation, where the validation set was used to find the optimal hyperparameters of the classifiers using grid search as well as the best model combination using the F1-score. See Supplementary Tables 3 and 4 for the hyperparameters space used during the fivefold cross-validation grid search and the best hyperparameters selected from the grid search.

### Feature analysis

To understand which features are important in improving the classification performance, we performed sequential backward feature selection (SBFS)^55^, a specific form of recursive feature elimination (RFE). SBFS attempts to reduce the number of feature spaces to a smaller size by sequentially removing features until the best subset of features that is most relevant to the prediction is obtained. We applied the SBFS to the six best models selected from the model selection pipeline above and chose the optimal subset of features based on the smallest subset of features within one standard deviation of the best cross-validation performance.

### Time-series data cleaning

We converted the pruned ALSPAC dataset to a time-series format. We first unified similar features’ coding to have the same meaning across all the time points at which they were recorded, such that they could be part of the same time series. Even if a feature was recorded for only one time point, we still treated it as a time-series feature. We also removed 40 features averaged across multiple time points and removed one cross-sectional duplicate variable. After this processing, we had 380 time-series features, which consisted of 377 independent features and three features for the participant identification (ID), timestamp, and target feature.

To reduce the feature space, we calculated a *t*-test between the depressed and not depressed samples for each feature and time stamp. If an independent feature did not have a *p*-value of < 0.05 for at least one of its time stamps, the feature was not included in the dataset. For the 12 through 18 age group, we removed 68.4% of the features. In this work, we refer to these datasets prepared for the 12 through 18 age group as *Dep12-18TS* (1799 samples, 120 features). See Supplementary Spreadsheet 2 for more details. The remaining data preprocessing steps were the same as that of the cross-sectional analysis.

### Time-series model selection

To select the best model configuration for predicting depression from the time-series data, we designed a time-series model selection pipeline to find the optimal preprocessing options and classifier, similar to the cross-sectional depression prediction. The pipeline was developed primarily using the scikit-learn^60^, imbalanced-learn^61^, MLxtend^62^, PyTorch^76^, and skorch^77^ Python packages. The time-series pipeline followed the same overall steps as the cross-sectional pipeline, which were preprocessing and classification. During the preprocessing step, the pipeline included three MVI methods (Last Observation Carried Forward (LOCF)^78^, Next Observation Carried Backwards (NOCB)^78^, Simple Imputer^79^), a categorical encoding method (one-hot encoding), and two FS methods (standard^63^, standard by sample) in the order listed. Since features in our time-series data were not measured frequently, we decided to skip outlier detection because many time-series outlier detection methods rely on data from neighboring time points for detection and smoothing. For additional information on how MVI methods were modified, see Supplementary Information 1.2.

For classification, we tested recurrent neural network (RNN)^80,81^ and Long Short-Term Memory (LSTM)^82^ with random resampling to upsample the minority class while training and a grid search to find the optimal hyperparameters. For each dataset, we randomly selected 20% for a held-out test set. The rest was used for fivefold cross-validation, where the validation set was used to find the optimal hyperparameters of the classifiers and the best model combination using the F1-score using grid search. See Supplementary Tables 5 and 6 for the hyperparameter space used during the fivefold cross-validation grid search and the best hyperparameters selected from the grid search.

### Significance statement

Depression is a major cause of disability and mortality for young people worldwide. Although depression is typically first diagnosed during adolescence, this outcome results from a developmental process that begins many years prior, as early as the prenatal period for some children, creating opportunities for early identification of children at risk. Unresolved questions are whether depression can be accurately predicted early in life and what factors are most predictive. The current study used ALSPAC data from the prenatal period to age ten years for 8467 participants to predict depressed status between ages 12–18 years. Overall prediction accuracy was 64%, and female sex, parental depression and anxiety, and exposure to stressful events or environments were identified as leading predictors of adolescent depression.

## Results

### Descriptive statistics

**Fig. 2:**
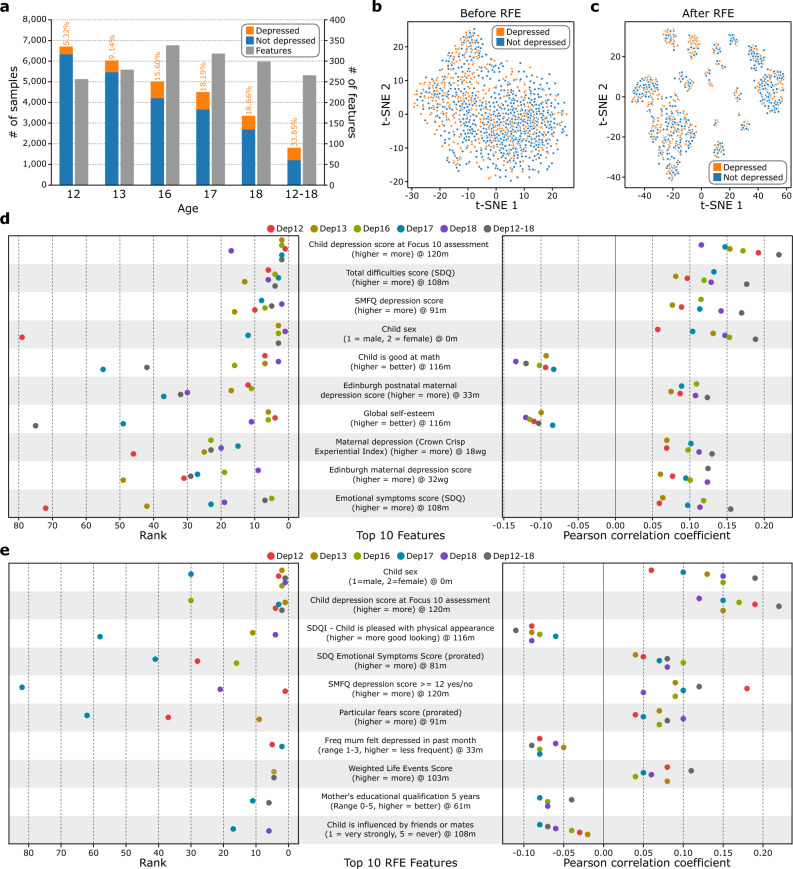
Statistics of the preprocessed ALSPAC data. (**a**) Number of participants and features for each derivative dataset generated from ALSPAC (Dep12, Dep13, Dep16, Dep17, Dep18, Dep12-18, from left to right). The percentages of depressed participants for each dataset are also on the graph in orange. (**b**) t-distributed Stochastic Neighbor Embedding (t-SNE) plot of the Dep12-18 data where all 266 independent features were used to generate the plot. (**c**) t-SNE plot of the Dep12-18 data where only the subset of 14 features after performing recursive feature elimination (RFE) was used to generate the plot. (**d**) Top 10 features (from top to bottom) highly correlated to the target variables. For each dataset, we calculated the Pearson correlation between the independent features and the target variable. We then assigned a rank to each feature, where the feature with the highest absolute correlation coefficient was assigned rank 1. We then averaged these ranks across all 6 datasets and identified the top 10 features (left). The respective correlation coefficient is shown on the right. The box represents the interquartile range, the middle line represents the median, the whisker line extends from minimum to maximum values, and the diamond represents outliers. The colored circles denote the raw data points (n = 6, 6 datasets). SDQ stands for the Strengths and Difficulties Questionaire while SMFQ stands for the Short Mood and Feelings Questionnaire. For the characters indicating age, w indicates weeks, m indicates months, and g indicates gestation. One duplicate feature was removed from the plot (Supplementary Information 1.1). (**e**) Top 10 features (from top to bottom) identified after performing RFE. For each dataset, we performed the model selection pipeline and performed RFE on the best model pipeline. Then, we ranked the RFE selected features according to the RFE results and sorted the features according to their number of appearances across the 6 datasets (e.g., # of appearance = 6 means that this feature was selected by RFE for all 6 datasets). The features that share the same # of appearances were further sorted incrementally by their average rank to identify the top 10 features (left). We also included the Pearson correlation from these features in the training data for all 6 datasets (right). The box represents the interquartile range, the middle line represents the median, the whisker line extends from minimum to maximum values, and the diamond represents outliers. The colored circles denote the raw data points (n = 6, 6 datasets), however, some features do not have data points for all datasets due to data preprocessing. SDQI stands for the Self Description Questionnaire-I. Freq stands for frequency. One duplicate feature was removed from the plot (Supplementary Information 1.1).

The number of participants with depression data in the dataset was 6715 at the age of 12 and it dropped to 3334 at the age of 18 due to missing data, while the number of independent variables included ranged from 255 at the age of 12 to 338 at the age of 16 (Supplementary Table 1, Fig. 2a). The class distribution in the datasets was unbalanced, such that the prevalence of depression was as little as 5.3% at age 12 and 33.9% when considering youth who were above the clinical cutoff for at least one time point between age 12 and age 18 (Fig. 2a). This prevalence is comparable to other estimates of the lifetime prevalence of depression obtained with self-report mental health questionnaires of participants from the UK Biobank study (29 to 35% depending on the measure, definition, and subsample used)^83^. The application of RFE for feature selection provided better visualization of the underlying structure of the adolescent population, as the t-SNE shows the Dep12-18 data forms groups after RFE that were relevant to depression prediction (Fig. 2b,c, Supplementary Fig. 8, Supplementary Spreadsheet 3).

### A rich repertoire of diverse features correlates with depression

In this work, we predicted adolescent depression from ALSPAC data. We generated six cross-sectional datasets from the ALSPAC dataset for each target age (predicting depression at ages 12, 13, 16, 17, and 18 years using features from the prenatal period to age ten entered simultaneously) and all target ages combined (predicting depression anytime between ages 12 to 18) by cleaning the feature set and keeping only the features correlated to the target variable (*p*-value < 0.05, see Methods). To identify the features consistently correlated with depression, we calculated the Pearson correlation coefficient between the independent and dependent variables for each of the six datasets. Finding the features that ranked at the top across all the datasets revealed that the child-reported depression score at age 10 was most strongly correlated to predicting depression across all ages (ρ = 0.17 ± 0.03, *p*-value < 5.55 × 10^–9^) (Fig. 2d), while the Strengths and Difficulties Questionnaire (SDQ) total difficulties score at 108 months, which is a composite measure of multiple behavioral and emotional indices including hyperactivity, emotional symptoms, conduct problems, and peer problems, ranked as the second most important feature (ρ = 0.12 ± 0.03, *p*-value < 1.76 × 10^–8^) (Fig. 2d). Next, the child’s SMFQ depression score reported at 91 months was positively correlated with depression (ρ = 0.12 ± 0.03, *p*-value < 1.07 × 10^–7^), females were more likely to have depression than males (ρ = 0.13 ± 0.04, *p*-value < 1.76 × 10^–8^), low math abilities were associated with greater likelihood of depression (ρ = − 0.10 ± 0.02, *p*-value < 4.90 × 10^–6^), a child whose mother had a higher Edinburgh postnatal maternal depression score when the child was 33 months was more likely to have depression (ρ = 0.10 ± 0.02, *p*-value < 2.57 × 10^–6^), a child with higher self-esteem was less likely to have depression (ρ = − 0.11 ± 0.01, *p*-value < 8.95 × 10^–5^), a child whose mother reported high maternal depression on the Crown Crisp Experiential Index (CCEI) at 18 weeks gestation was more likely to have depression (ρ = 0.10 ± 0.02, *p*-value < 1.61 × 10^–6^), a child with a mother who had a high Edinburgh maternal depression score at 32 weeks gestation was more likely to have depression (ρ = 0.10 ± 0.02, *p*-value < 2.64 × 10^–5^), and a child who scored higher on the difficulties score from the SDQ at 108 months was more likely to have depresssion (ρ = 0.10 ± 0.03, *p*-value < 2.42 × 10^–5^) (Fig. 2d). The ranked lists of the features assigned using the Pearson correlation coefficient for each dataset are similar to each other when compared to the random baseline (rank-based overlap (RBO)^84^ = 0.48 ± 0.08 vs. 0.10 ± 0.02, respectively, *p*-value = 2.87 × 10^–16^; Supplementary Information 1.3).

### Adolescent depression can be predicted but with low confidence

**Fig. 3:**
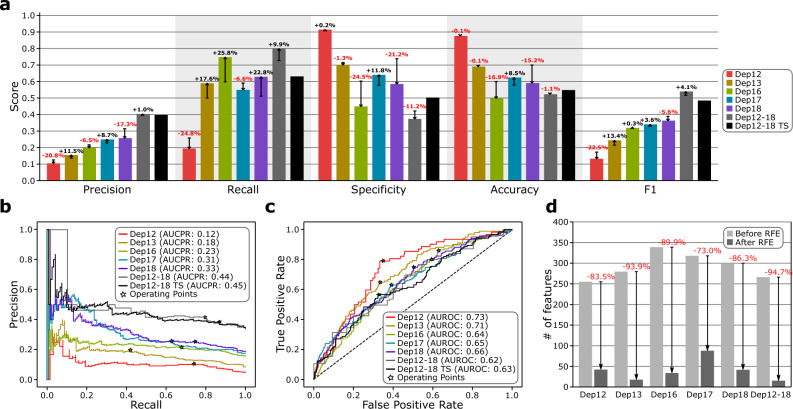
Performance and evaluation of the machine learning models. (**a**) Precision, recall, specificity, accuracy, and F1-score for the 6 best performing models for each cross-sectional dataset after performing the RFE and for the best-performing time-series model for the Dep12-18TS dataset. The whisker line and the corresponding percentage value denote the change in score for the cross-sectional models after the RFE was performed. (**b,c)** The precision-recall (PR) and receiver operating characteristic (ROC) curves of the 6 best performing models for each cross-sectional dataset after the RFE and of the best-performing time-series model for the Dep12-18TS dataset obtained from the held-out test set. The operating point was selected using the F1-score. The area under the precision-recall curve (AUCPR) and the area under the receiver operating characteristic curve (AUROC) are shown in the legends. See Supplementary Figs. 9 and 10 for PR and ROC curves before RFE and Supplementary Figs. 11–16 for the RFE curves. **(d)** Number of features before and after running the RFE for each cross-sectional dataset.

We ran the model selection pipeline to select the best combination of preprocessing steps (feature scaling, missing value imputation, and outlier detection) and binary classifiers for all six datasets, as well as the RFE (see Methods and Fig. 1). Evaluation of these model selection pipelines, which have been optimized for the F1-score using a fivefold cross-validation and held-out test set, showed that robust for FS (4 out of 6), either KNN or MICE for MVI (3 for each), either none or LOF for OD (3 for each), and MLP for the classifier (4 out of 6) were the best combination (Supplementary Table 7). The prediction performance increased as the target age increased, with an F1-score of 0.13 at age 12 to 0.37 at age 18 (173.9% increase), while the baseline (always predict ‘depressed’) was 0.09 and 0.32, respectively (Fig. 3a). We obtained the best F1-score of 0.54 for the merged dataset Dep12-18 (baseline 0.52). It is possible that this trend occured because the class imbalance between depressed and not depressed samples decreases as age increases, which reduced the amount of up-sampling occurring (Fig. 2a). The best-performing models for all datasets also outperformed their respective baselines in both the area under the precision-recall curve (AUCPR) and the area under the receiver operating characteristic curve (AUROC) (Fig. 3b,c; Supplementary Tables 8–13). The time-series model optimized for the Dep12-18TS data (standard for FS, NOCB for MVI, and RNN for classifier) did not perform better than the baseline nor the best cross-sectional F1 score (0.49, 0.51, and 0.54, respectively), although the models AUCPR and AUROC were better than the baseline (see Fig. 3 and Supplementary Tables 13 and 14). See Supplementary Fig. 17 and Supplementary Information 1.4 for precision-recall (PR) and receiver operating characteristic (ROC) curves when taking a subset of the features of the Dep12-18TS data.

### A few features are adequate to predict adolescent depression

Figure 2e depicts the top features for each model, with “Child sex” and “Child depression score at Focus 10 assessment” being some of the top features, consistent with the univariate analysis (Fig. 2d). Through RFE, we were able to significantly reduce the feature size from 73.0% (318 to 86) for Dep17 up to 94.7% (266 to 14) for Dep12-18 (Fig. 3d) with insignificant changes to all of the test metrics before and after the application of the RFE (precision *p*-value = 4.6 × 10^–1^, recall *p*-value = 1.9 × 10^–1^, specificity *p*-value = 2.4 × 10^–1^, accuracy *p*-value = 3.2 × 10^–1^, and F1-score *p*-value = 9.8 × 10^–1^; Supplementary Tables 8–13). Although the ranked lists of the remaining features after the RFE for each dataset were similar to each other when compared to the random baseline (RBO = 0.14 ± 0.06 vs. 0.10 ± 0.06, respectively, *p*-value = 3.30 × 10^–2^; Supplementary Information 1.3), only child sex appeared in all six post-RFE subsets, whereas child-reported depression score at age 10 appeared in all subsets except for Dep18 (Fig. 2e, Supplementary Table 15, Supplementary Fig. 18, Supplementary Spreadsheet 4)*.* Interestingly, the RFE-ranked subfeatures as a whole were not correlated with the features ranked using the Pearson correlation coefficient for any of the datasets ($\tau$ = 0.04, *p*-value = 3.51 × 10^–1^ for Dep 12; $\tau$ = 0.05, *p*-value = 2.00 × 10^–1^ for Dep 13; $\tau$ = − 0.02, *p*-value = 5.22 × 10^–1^ for Dep 16; $\tau$ = 0.04, *p*-value = 2.50 × 10^–1^ for Dep 17; $\tau$ = 0.02, *p*-value = 6.70 × 10^–1^ for Dep 18; $\tau$ = − 0.01, *p*-value = 8.14 × 10^–1^ for Dep 12–18). Comparing the lists of the top five predictors identified with RFE for each age when the depression outcome was measured revealed both common predictors across different ages and predictors that were more influential in predicting depression at a specific age (see Supplementary Table 16). Female sex and various indices of maternal depression in childhood were among the top five predictors for depression at multiple ages. The quality of the relationship with parents at age 10 was among the top five predictors for depression at age 12 but not for any later time points. Childhood problems with peers and not being good with athletics were among the top five predictors for depression at age 13 but not other ages. Participants’ scholastic competence score and teacher ratings of their general knowledge in childhood were among the top five predictors for depression at ages 16 and 18, respectively.

## Discussion

Our study revealed multiple statistically significant associations of early life environmental, socio-emotional, cognitive, and biological variables with adolescent depression, several of which lend empirical support, based on a large sample and numerous variables, to the stress accumulation and stress sensitization theories of depression^7,8^. Although we predicted adolescent depression with adequate performance, we were unable to reach the recommended level for high clinical relevance as it has been proposed that precision of 80% or more would be necessary to reach high clinical relevance (i.e., if 80% of patients predicted to have a disorder using a prediction algorithm would be true positives according to subsequent diagnosis)^85^. This may relate to this dataset being collected from a community sample as opposed to a clinic-referred cohort. While we used time-series models like RNN and LSTM, we did not observe a performance gain over the cross-sectional models, which we hypothesized to be due to the sparsity of the dataset with many missing values, such that 38.8% of the features (343 out of 885) have more than 50% missing values. As a whole, the large amount of missing data and the limitations of imputation procedures may have also constrained the prediction performance of the final models.

We identified a subset of features that carried more predictive power for each derivative dataset and are consistent with prior theory and empirical research. Female sex was a leading feature across all models, consistent with the 2:1 female-to-male prevalence ratio found in epidemiological studies^86–88^ and emerging research about the role of the estrogen receptor in depression^89^. Parental depression and anxiety also emerged as leading features (e.g., Edinburgh postnatal depression scores at 32 weeks gestation and 33 months postnatal and maternal depression as measured on the CCEI, measured at 18 weeks gestation from the top 10 Pearson correlated features (Fig. 2d), and frequency the mother was depressed when the child was aged 33 months from the top 10 RFE features (Fig. 2e)), consistent with a prior study using ML to predict depression in youth using the Adolescent Brain Cognitive Development dataset^90^ and other studies on the genetic and environmental impacts of being raised by parents who suffer from mood and anxiety disorders^91,92^. Furthermore, other recent work using ML found that the parental emotional state was an important predictor of depression trajectories in early adolescence^48^. Consistent with leading theories of depression as a disorder of stress accumulation and stress sensitization^7,8^ and prior empirical evidence^48^, features indicating exposure to stressful events or stressful environments (financial hardship, parental unemployment, poor neighborhood quality, child life events, etc.) emerged as important in our analysis (e.g., SDQ total difficulties score at 108 months from the top 10 Pearson correlated features (Fig. 2d); weighted life events score at 108 months from the top 10 RFE features (Fig. 2e); variables related to job and employment status of the mother and partner being selected by RFE for all except Dep12 (Supplementary Spreadsheet 4); and variables related to the mother and partner’s opinion of the neighborhood being selected by RFE for Dep17 and Dep12-18 (Supplementary Spreadsheet 4)). The predictive value of composite variables such as child life events scores supports stress accumulation theories of depression, given that children who accumulated more adverse events were more vulnerable to depression during adolescence. Additionally, the predictive value of early-life variables, such as maternal perceived social support during her child’s infancy, provides support for early-life stress sensitization theoretical models, which postulate that stressful early-life experiences can leave an imprint on subsequent developmental trajectories.

In addition, we found that some predictors were influential in predicting depression outcomes at multiple ages across adolescence, whereas others were especially salient in predicting depression at a specific age (Supplementary Table 16, Supplementary Fig. 18). For example, female sex was among the top five predictors for depression at ages 12, 13, 16, 18, and of suffering from depression for at least one time point in the 12–18 age range, suggesting girls have heightened vulnerability across all of adolescence. However, the quality of the relationship with parents only appeared among the top five predictors when predicting depression at age 12, but not at later ages, which may be explained by developmental trends towards increasing independence from parents and increased reliance on peers across adolescence^93,94^. Consistent with the puberty-related shift from relying on parents to relying on peers, the quality of peer relationships rose among the top five predictors of depression at age 13. Scholastic competence and teacher ratings of general knowledge in childhood rose among the top five predictors for depression at ages 16 and 18, when youth were approaching the end of high school or preparing for university entry, suggesting that a history of academic difficulties may be particularly influential for depression risk around the end of high school. If replicated by future work, these results suggest possible avenues for age-specific prevention and intervention strategies to ameliorate adolescent depression.

Predicting the onset of depression among adolescents is a challenging task because humans function as open dynamic systems in a constant transaction with and adaptation to the environment, and therefore, long-term future outcomes may be causally indeterminate and remain open to multiple developmental pathways at any given time^95,96^. This may be particularly true when forecasting adolescent outcomes, given that adolescence has been recognized as a developmental period with heightened neural and behavioral plasticity and more openness to the environment compared to other periods^97,98^. Thus, outcomes during this period may be inherently less predictable than outcomes during adulthood^40^. Additionally, longitudinal studies on the development of psychopathology, including depression, have also shown substantial evidence of equifinality (individuals can develop the same disorder despite having different developmental histories) and multifinality (individuals can develop different disorders or no disorders despite having similar developmental pathways and growing up in the same homes)^99^. Equifinality and multifinality pose significant challenges to long-term prediction from prior developmental variables and may highlight the role of stochastic events in development. Stochastic events may make prediction difficult at the individual level, even when research has identified a large number of useful predictors at the population level^100^. Generally, ML studies have higher prediction accuracy if conducted with adults^40^, or if they use ML to detect current depressed status^106^ or aim for short-term prediction^101^ than studies with adolescents aiming for long-term prediction^47^.

One potential solution for improving prediction accuracy in future studies would be to use recently obtained features (as opposed to past or chronic life events) to predict depression over shorter time frames (e.g., 1 month or 3 months prior). Recent findings^8,101^ suggest that combining information on childhood developmental history (e.g., data from gestation to age 10 years as included in the current project) with information about recent acute stressors in adolescence (e.g., from the previous 0–3 months) may be necessary to achieve high accuracy for predicting the onset of depression, though this possibility would limit our ability to implement interventions in a timely fashion several months or years before the onset of depressive symptoms or disorder. Incorporating more in-depth data on stress physiology (e.g., multiple days of cortisol output or hair cortisol measures) may also improve prediction accuracy given prior meta-analytic evidence linking depression to elevated cortisol output^107^. In addition, the missing data due to attrition over time is a well-recognized challenge in human longitudinal research and is also a limitation of the current cohort study, as it can introduce selection bias. Nevertheless, depression prevalence rates at age 18 in this study were comparable to other reports from studies in the UK, suggesting that results may generalize. Future research should incentivize participant retention. To improve the prediction accuracy, our next steps are to further extend the results by updating our model selection pipeline with additional tools and incorporating multimodal neurobiological^48,102–104^ and genomic features. Whether diversifying the data sources and reducing data missingness will improve prediction accuracy for distant psychiatric outcomes remains to be determined. Because ML methods have provided useful insights in a number of biomedical fields, future research should aim to examine methods of improving prediction accuracy for psychological outcomes. If short- and long-term individualized prediction proves difficult with current methods and assessment tools, cost-effective universal prevention programs may be delivered to all children (e.g., via schools) as an alternative or complementary prevention strategy.

## Conclusions

Although prediction performance did not reach clinical relevance in the current study or in other recent attempts to use developmental datasets to predict long-term psychological or behavioral outcomes^47,105^, this study provides insight into additional approaches that might be successful. Additionally, the predictors influencing depression prediction from our models were consistent with previous work. The leading informative features in our predictive models of adolescent depression were sex, parental depression and anxiety, and exposure to stressful events or environments. This study demonstrates how using a broad array of evidence-driven predictors from early in life can inform the development of preventative decision support tools to assist in the early detection of risk for mental illness.

## Supplementary Information

Supplementary Information 1.

Supplementary Information 2.

Supplementary Information 3.

Supplementary Information 4.

Supplementary Information 5.

## Data Availability

All code and instructions on how to reproduce the results can be found at https://github.com/IBPA/DepressionProject.
