# Supplementary material for: Prediction of adolescent depression from prenatal and childhood data from ALSPAC using machine learning
Source: Sci Rep. 2024 Oct 7;14:23282. doi: 10.1038/s41598-024-72158-9 (PMC11458604; doi:10.1038/s41598-024-72158-9)
Supplement: Supplementary file 5 — Supplementary Information 5. [file 41598_2024_72158_MOESM5_ESM.docx]

***Prediction of Adolescent Depression from Prenatal and Childhood Data from ALSPAC Using Machine Learning.***

Arielle Yoo^1,2,3#^, Fangzhou Li^1,2,3#^, Jason Youn^1,2,3#^, Joanna Guan^4^

Amanda E. Guyer^5^, Camelia E. Hostinar^4^, and Ilias Tagkopoulos^1,2,3*^

^1^ Department of Computer Science, University of California – Davis

^2^ Genome Center, University of California – Davis

^3^ USDA/NSF AI Institute for Next Generation Food Systems (AIFS)

^4^ Department of Psychology, University of California – Davis

^5^ Department of Human Ecology, University of California – Davis

^#^ Equal contribution.

Corresponding author: *[itagkopoulos@ucdavis.edu](mailto:itagkopoulos@ucdavis.edu)

SUPPLEMENTARY INFORMATION

Table of Contents

[1 Supplementary Text 3](#_Toc171000355)

[1.1 Data Cleaning 3](#_Toc171000356)

[1.2 Time Series Missing Value Imputation Method Adjustments 4](#_Toc171000357)

[1.3 Comparison of ranked lists of features 5](#_Toc171000358)

[1.4 A smaller subset of features does not improve time-series performance. 5](#_Toc171000359)

[1.5 Additional Discussion 6](#_Toc171000360)

[2 Supplementary Figures 10](#_Toc171000361)

[3 Supplementary Tables 28](#_Toc171000362)

[4 References 50](#_Toc171000363)

# Supplementary Text

## Data Cleaning

To prune the dataset from 6,163 features to 885 features, research assistants inspected each variable and referred to the data dictionary to organize the dataset. We organized the dataset as follows: 1) single variables that represent single questions from questionnaires were combined into an average or sum score variable for the questionnaire and individual questions were not used if they were part of a scale (e.g., we used subscale scores for the infant temperament dimensions of Activity, Rhythmicity, Approach, and Adaptability instead of using individual questions making up these scales); 2) variables over age 10 were not relabeled unless they were depression scores because we did not use variables above age 10 for prediction; 3) when both the mother and partner reported the same information (e.g., family income), the mother report was used and the partner report was dropped because mother reports had larger sample sizes; 4) when different sum scores were provided for the same scale by the ALSPAC team (e.g., a score for complete cases and a prorated score, or a score corrected for gestational age and an uncorrected score), we only used one score recommended by Avon Longitudinal Study of Parents and Children (ALSPAC) for use, in order to be consistent with prior publications; 5) when information was provided at different ages was not likely to change (e.g., child race/ethnicity), we used the first timepoint only; and 6) when a variable was a follow-up question to giving one specific answer to a previous question, the follow-up question was dropped because few participants had answers to the follow-up question. For example, we removed a variable asking the number of weeks since the mother’s last marriage if there was a marriage in the past year, and, although we included items regarding children having special education needs, we did not include answers to follow-up questions about whether a child with special educational needs was in a hospital school (no participants chose “yes”, all participants had “no” for this item) or was home-schooled (only n = 2 participants had a “yes” for this question, all others had “no”, which did not provide enough variability on this follow-up question or sufficient sample size for each category to estimate effects).

During data cleaning, one feature was inadvertently duplicated (y10CH_Dep_127m and fddp130_120m). This feature was removed from Figure 2e, d plots such that only fddp130_120m is shown. However, both features can still be seen in supplementary material and the rest of our work.

## Time Series Missing Value Imputation Method Adjustments

For the missing value imputation (MVI) methods, we had to modify them slightly so that they would work for samples that are missing data for entire features. For Last Observation Carried Forward (LOCF), first, we use the last observation’s value to fill in the future time points for that feature, then for edge cases where there is no last observation we use the normal Next Observation Carried Backwards (NOCB) process, and finally for samples that are empty for the feature, we use a dataset-wise simple imputer to fill in the mean value for numerical features or mode value for categorical features from the entire dataset. For NOCB we conduct a similar process where first we use the next observation’s value to fill in the past time points for that feature, then we conduct the normal LOCF process, and finally, we use a dataset-wise simple imputer. For simple imputer, we first do the normal simple imputer process where we use the sample’s mean value for numerical features or mode value for categorical features to impute, and then we do the dataset-wise simple imputer to fill in the remaining values.

## Comparison of ranked lists of features

To check how the ranked lists of the features assigned using the Pearson correlation coefficient for each dataset are similar to each other when compared to the random baseline, we used rank-based overlap (RBO)(1,2) which checks if two ranked lists are in agreement. RBO value ranges between 0 and 1, where 0 means complete disagreement and 1 means a complete agreement between the two ranked lists. RBO allows comparing two disjoint ranked lists of different lengths as well as putting more emphasis on the agreement at the top of the lists. To this end, we first ranked the features from most important (top) to least important (bottom) for each dataset (**Supplementary Spreadsheet 2**). We then calculated the RBO values of all 15 pairwise combinations of the 6 datasets. For the baseline, we similarly calculated the RBO values but after randomly shuffling the order of the features. We finally compare these two lists of RBO values to report the statistics (RBO = 0.48 ± 0.08 vs. 0.10 ± 0.02, respectively, *p*-value = 2.87x10^-16^). Note that this process was applied identically to the post-RFE features and duplicate features were removed.

## A smaller subset of features does not improve time-series performance.

Using the 14 cross-sectional RFE selected features for Dep12-18 data, we found the corresponding 13 time series features in the Dep12-18TS data since 1 cross-sectional feature was a time series feature recorded at a different time. Using this subset of the Dep12-18TS data which we call Dep12-18TS-CSRFE, we performed the same model selection pipeline to find the best combination of preprocessing steps (feature scaling (FS), MVI) and classifier. While optimizing for F1-score using 5-fold cross-validation, we find the best combination for preprocessing for Dep12-18TS-CSRFE is standard for FS and Simple for MVI, and the model used was long Short-Term Memory (LSTM). The F1 score from this best pipeline is 0.44, which is worse than the best F1 score without reducing features (0.49) and again worse than the baseline F1 score of 0.51. The area under the precision-recall curve (AUCPR) is also worse than baseline, and the area under the receiver operating characteristic curve (AUROC) is barely better than the baseline (see **Supplementary Table 13** and **Supplementary Figure 16**).

## Additional Discussion

Our prediction performance is consistent with a prior study using machine learning (ML) to predict whether youth had elevated levels of parent-reported mental health problems rated on the Strengths and Difficulties Questionnaire (SDQ) at age 15 based on features from ages 9 and 12 in The Child and Adolescent Twin Study in Sweden(3), which had a high recall of 0.91, but the specificity of detecting presence versus absence of mental health problems above a clinical cutoff that was too low to have clinical utility (0.30). Additionally, our results are consistent with results from the Fragile Families Challenge, a team science effort of 160 research teams using ML-based prediction models to predict six important life outcomes using 15 years of longitudinal data from the Fragile Families birth cohort study In the United States(4). The six life outcomes were child grade point average, child grit, household eviction, household material hardship, primary caregiver layoff, and primary caregiver participation in job training. Prediction accuracy across research teams, prediction models, and life outcomes was low and only slightly differed from benchmark models. The present study adds to evidence that the predictability of human psychological or behavioral outcomes is only slightly better than chance, even when using rich longitudinal datasets that assess multiple domains using multiple informants over long periods.

First, long-range forecasting of psychological outcomes 2 to 8 years later may not be possible with high accuracy if distant outcomes are at least partially statistically indeterminate. That is, having access to rich data about a child’s developmental history up to age 10 may not be sufficient to predict a psychological outcome at age 12 or 18. Pubertal onset also has the potential to reset developing systems and may alter the influence of earlier life influences on later outcomes. Indeed, puberty signifies the opening of a sensitive period for experience-dependent neural plasticity intended to facilitate cognitive, emotional, and social maturation(5).

Recent advances in forecasting methodology for other complex systems (e.g., data assimilation techniques that dynamically and continuously adjust prediction models for weather forecasts based on new incoming information)(6) and testing alternate conceptual perspectives that help increase the explanatory power of complex and indeterminate phenomena(7) may prove fruitful in future psychological research. Prior literature has shown that stressful life events are a trigger for depression(8), though only a subset of the population develops depression when confronted with stressful life events(8). A recent meta-analysis showed that a fairly accurate prediction of postpartum depression in women can be achieved using ML applied to features assessed a few months before pregnancy (e.g., prenatal depression or anxiety), with the area under the receiver operating characteristic curve being above 0.70 across all 11 studies(9).

Prediction accuracy may also have been constrained due to the measurement of depression in this study. The presentation of depression is ripe with heterogeneity in adolescence due to variation in when depression onsets, the specific permutation of symptoms that manifest, the course depression takes over time, and the impact of potential comorbidities(10). The measure of depression available in this study reflected the degree to which children endorsed phrases regarding how they were feeling or acting within the last two weeks. Other measurement tools, such as a semi-structured psychiatric interview reviewed by a clinician for diagnostic determination (e.g., Kiddie Schedule for Affective Disorders and Schizophrenia (K-SADS)), may enhance the predictive accuracy of clinical depression. However, such interviews are prohibitively time and cost-intensive for use in very large samples and the Short Mood and Feelings Questionnaire (SMFQ) used in this study has excellent psychometric properties.

Although unavailable for inclusion in the current study, prediction accuracy may also be improved by incorporating neurobiological features that capture participants’ brain function and structure given robust evidence of depression-related disruptions and alterations in the brain in adolescence and adulthood(11,12) even prenatally(13). Results from the large Adolescent Brain Cognitive Development (ABCD) Study in the U.S. revealed some magnetic resonance imaging (MRI)-derived neurobiological markers that were among the top 10 leading predictors of four adolescent depression trajectory classes, though overall balanced accuracy for predicting these classes was only moderate (0.64)(14). Additionally, a small recent study of 33 participants suggested that cortical thickness estimated with MRI might be a useful predictor for developing adolescent depression within a 5-year follow-up window, which affected 18 of the 33 participants(15). However, this study found that reduced cortical thickness in the medial orbitofrontal cortex predicted depression, whereas a large prior study had linked depression risk to greater cortical thickness in this region, thus the inconsistent findings and small sample size suggest a need for further replication of these results. The high cost associated with MRI scans reduces the feasibility of using them for widespread screening at the population level, though it is possible these neurobiological markers would improve prediction accuracy as seen in the ABCD Study(14). Future research should examine additional behavioral tasks, survey measures, or other physiological markers that might be more affordable to collect than MRI while serving as good proxies for certain aspects of brain function ascertained using MRI.

Incorporating genomic data for participants may also improve prediction accuracy. The present study did not include genomic features in the prediction models but included data on the maternal and paternal history of depression and anxiety, which serve as proxies of genetic liability and exhibited small statistically significant associations with our depression outcome. Additionally, recent findings from the large United Kingdom (UK) Biobank study of half a million women and men revealed that SNP-based heritability estimates for depression were fairly low, ranging from 11% heritability for self-reported depression to 32% for strictly defined and recurrent (which tends to be more severe) depression(16). Thus, although genomic information may improve prediction accuracy, the small amount of variance explained by genetics in depression and our inclusion of family history predictors mitigates this limitation in the present study. Beyond the main effects of genes, future research should examine statistical interactions between genetic and environmental data to test whether their interaction yields more successful predictions.

There are potentially more improvements that can be done using models specifically designed with sparse, irregularly time-sampled data(17). We leave this for future work.

# Supplementary Figures


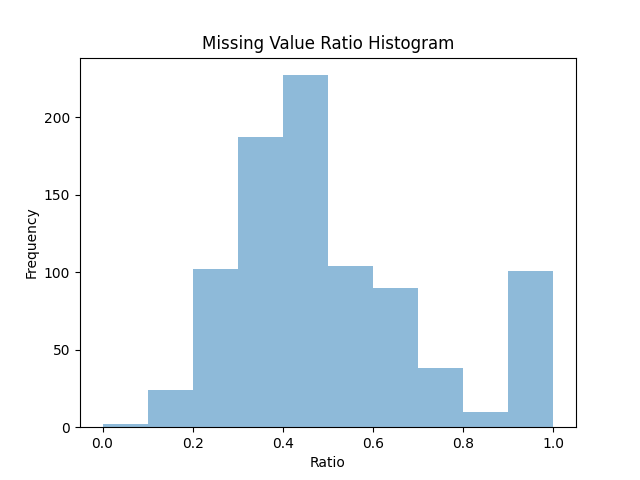


Supplementary Figure 1. Missing Value Ratio vs. Number of Features. Visualization of Supplementary Table 2. Frequency = the number of features, Ratio = number of samples without data / total number of samples


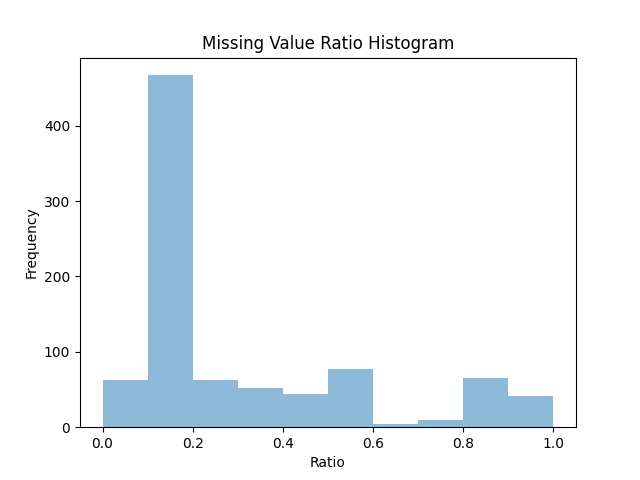


Supplementary Figure 2. Missing Value Ratio vs. Number of Features for Dep12. Frequency = the number of features, Ratio = number of samples without data / total number of samples


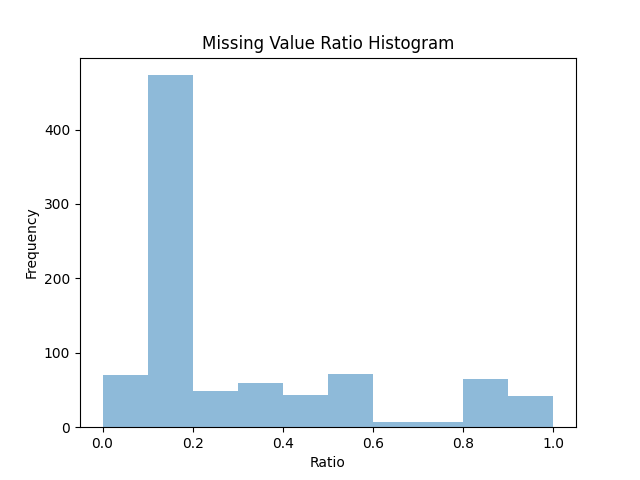


Supplementary Figure 3. Missing Value Ratio vs. Number of Features for Dep13. Frequency = the number of features, Ratio = number of samples without data / total number of samples


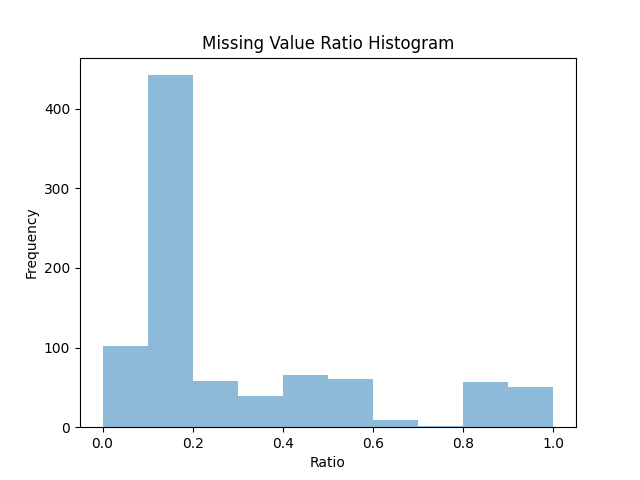


Supplementary Figure 4. Missing Value Ratio vs. Number of Features for Dep16. Frequency = the number of features, Ratio = number of samples without data / total number of samples


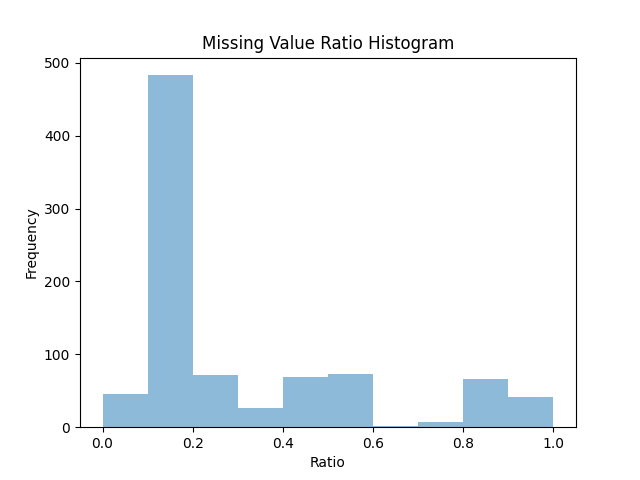


Supplementary Figure 5. Missing Value Ratio vs. Number of Features for Dep17. Frequency = the number of features, Ratio = number of samples without data / total number of samples


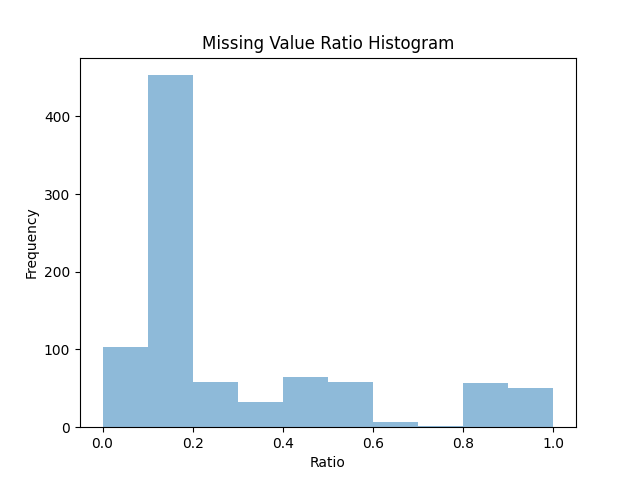


Supplementary Figure 6. Missing Value Ratio vs. Number of Features for Dep18. Frequency = the number of features, Ratio = number of samples without data / total number of samples


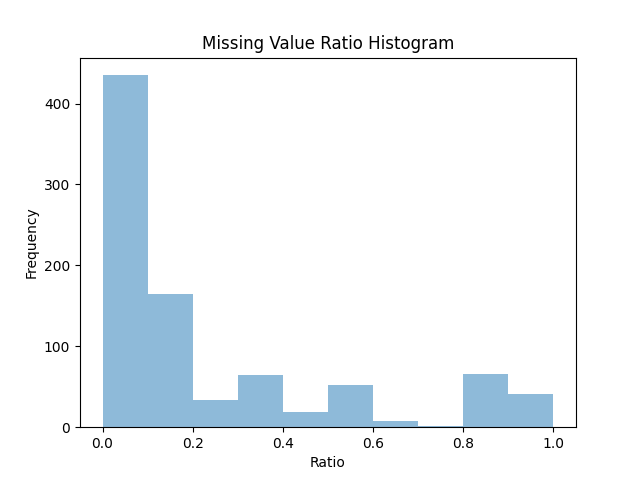


Supplementary Figure 7. Missing Value Ratio vs. Number of Features for Dep12-18. Frequency = the number of features, Ratio = number of samples without data / total number of samples


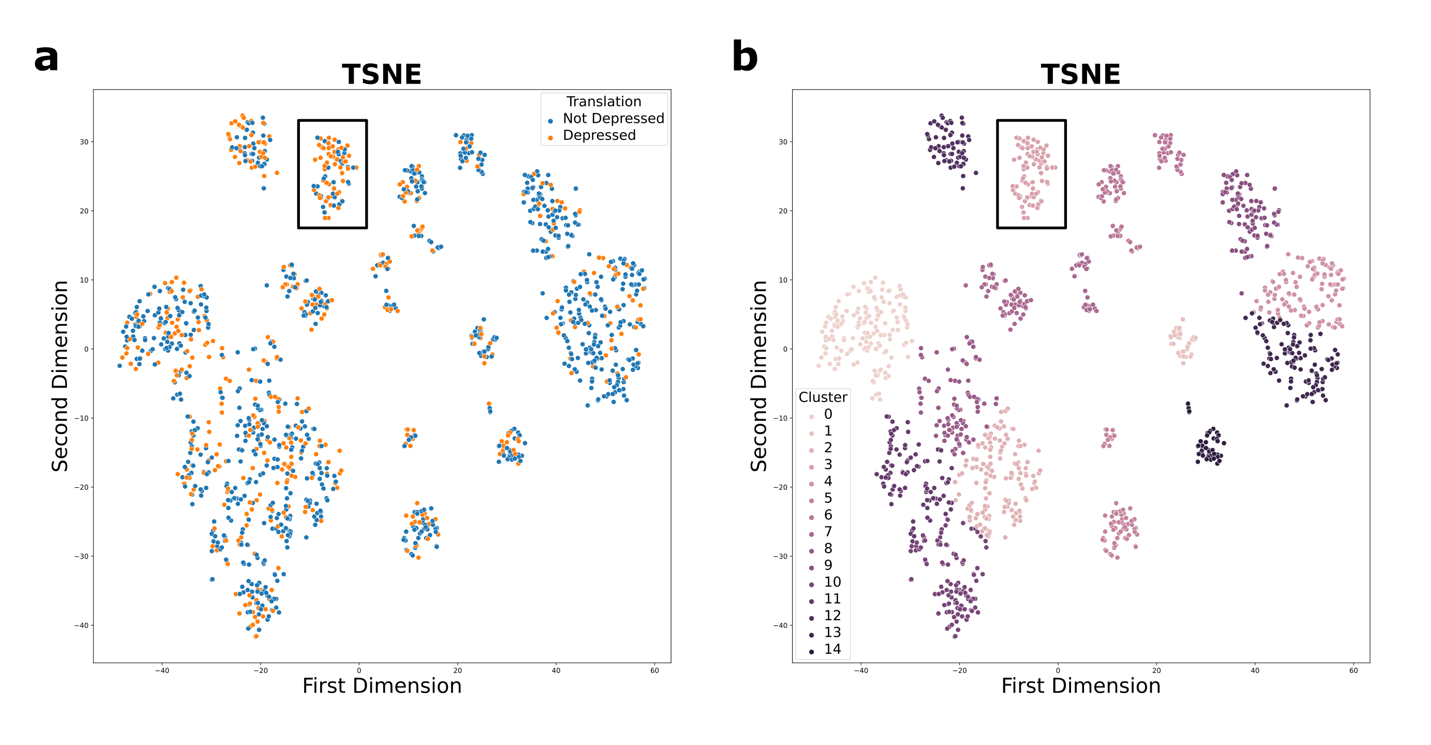


**Supplementary Figure 8. t-distributed Stochastic Neighbor Embedding (t-SNE) plot of the Dep12-18 training data after RFE selected 14 features with a black box around Cluster 3. This training data has undergone minmax feature scaling and KNN missing value imputation from the Dep12to18 model selection pipeline. a t-SNE plot of the Dep12-18 data after RFE color-coded by whether the sample was depressed or not. b t-SNE plot of the Dep12-18 data color-coded by K-means clustering results. Cluster 3 has a high ratio of depressed samples compared to other clusters and contains all female samples, which matches our results that female samples are more likely to be depressed. See Supplementary Spreadsheet 3 for additional feature information for cluster 3 compared to the other clusters and the overall training data after RFE.**


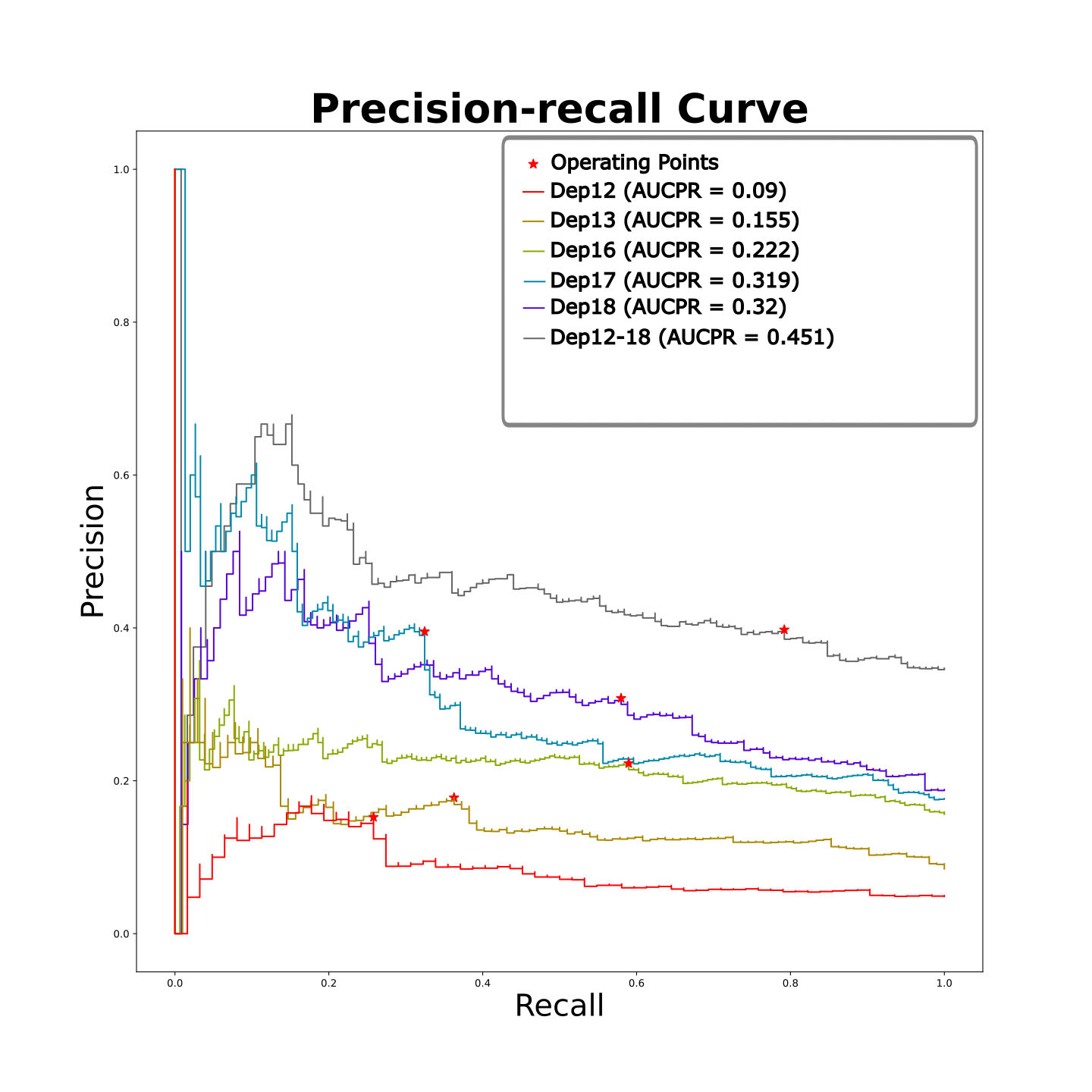


Supplementary Figure 9. Precision recall curve for all datasets’ best model pipeline before recursive feature elimination (RFE). Optimal Operating Point is where F1 score is highest


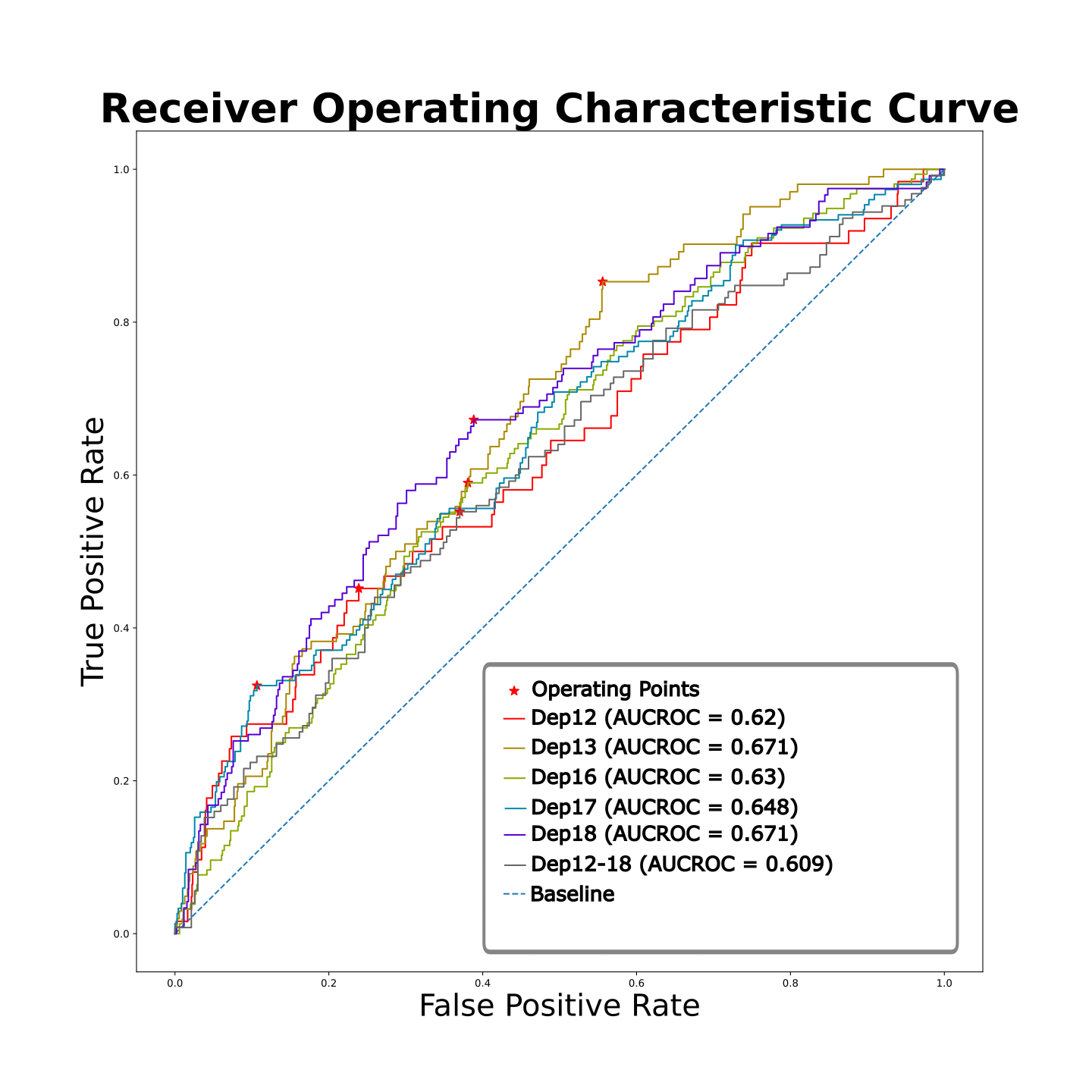


Supplementary Figure 10. Receiver operating characteristic curve for all datasets’ best model pipeline before recursive feature elimination (RFE). Optimal Operating Point is where the true positive rate – false positive rate is highest


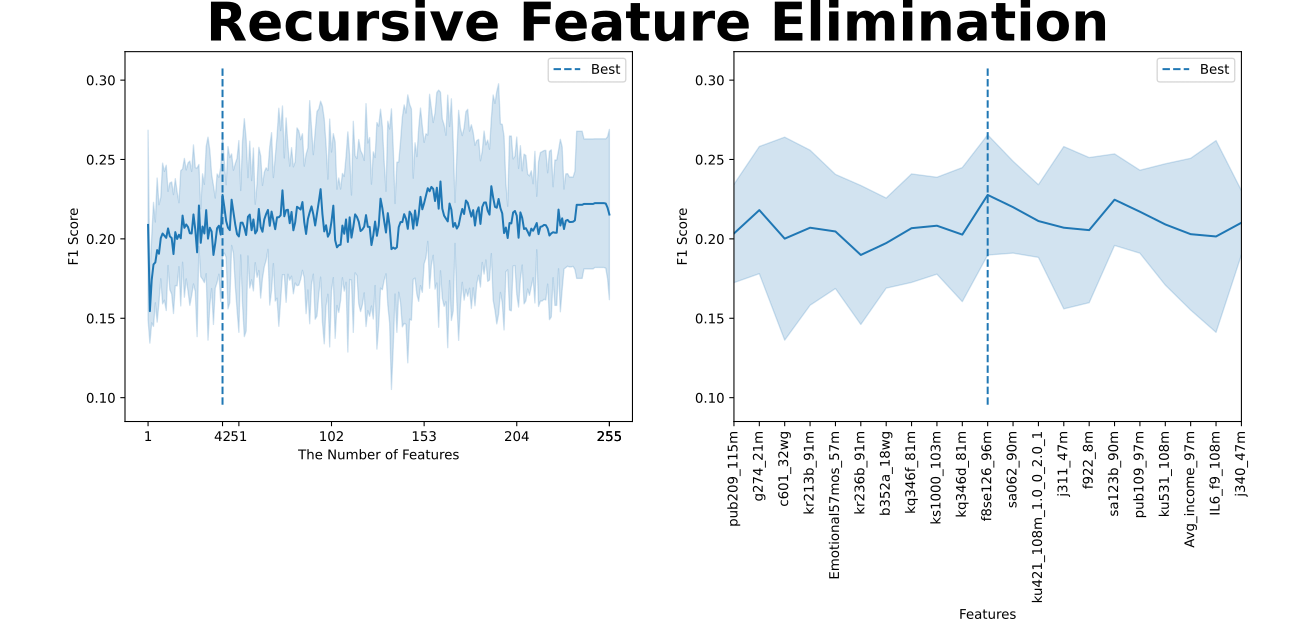


Supplementary Figure 11. Recursive Feature Elimination of 5-Fold Cross Validation of Dep12 for the best model pipeline (feature scaling (FS): robust, missing value imputation (MVI): k-Nearest Neighbors (KNN), outlier detection (OD): local outlier factor (LOF), classification (CLS): AdaBoost)


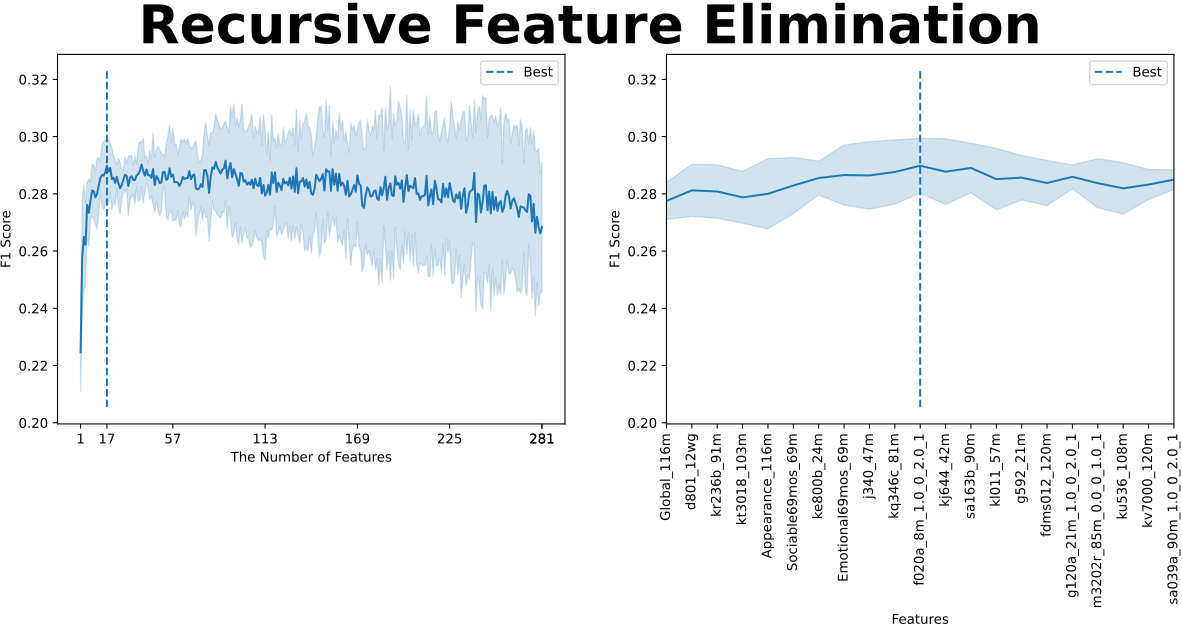


Supplementary Figure 12. Recursive Feature Elimination of 5-Fold Cross Validation of Dep13 for the best model pipeline (feature scaling (FS): robust, missing value imputation (MVI): Multivariate Imputation by Chained Equations (MICE), outlier detection (OD): None, classification (CLS): multilayer perceptron (MLP))


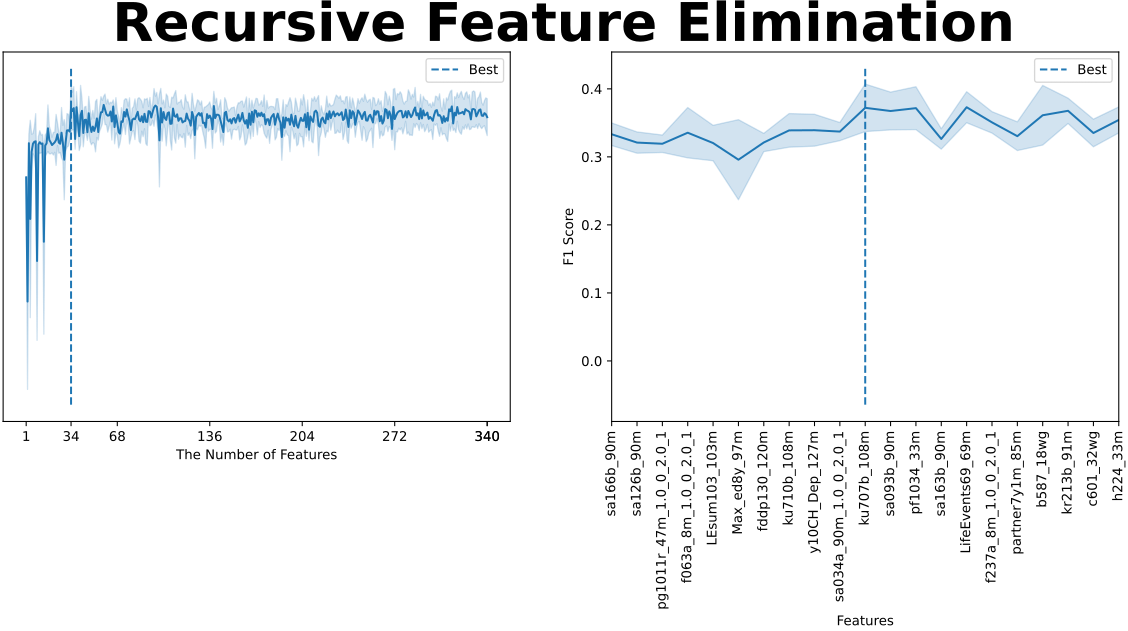


Supplementary Figure 13. Recursive Feature Elimination of 5-Fold Cross Validation of Dep16 for the best model pipeline (feature scaling (FS): minmax, missing value imputation (MVI): k-Nearest Neighbors (KNN), outlier detection (OD): None, classification (CLS): multilayer perceptron (MLP))


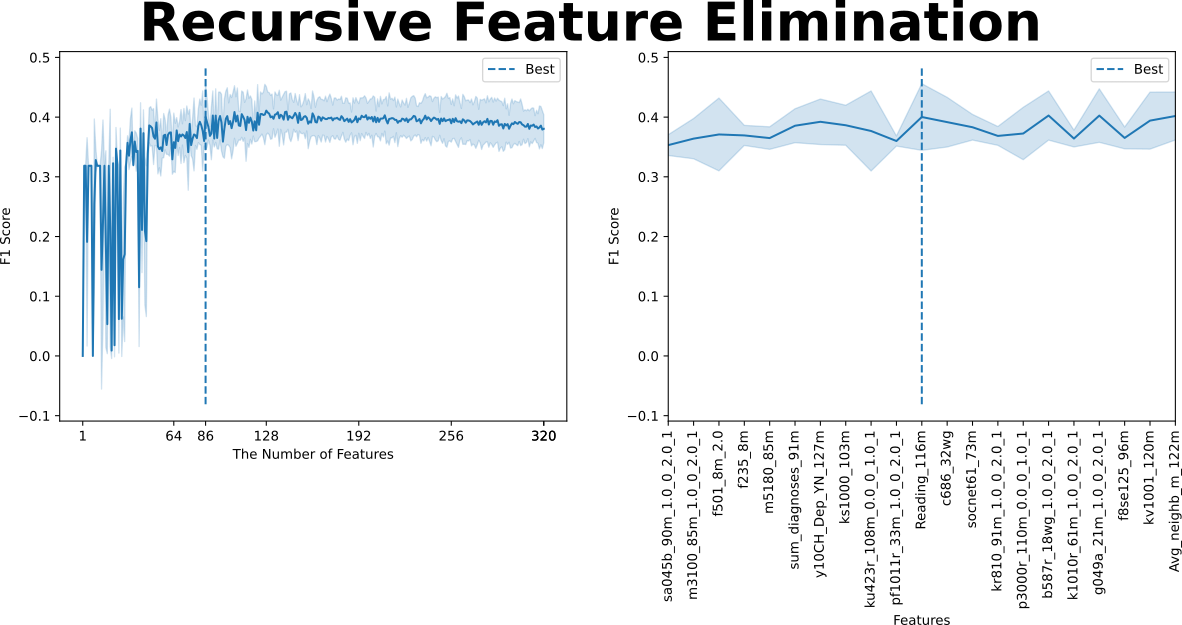


Supplementary Figure 14. Recursive Feature Elimination of 5-Fold Cross Validation of Dep17 for the best model pipeline (feature scaling (FS): robust, missing value imputation (MVI): Multivariate Imputation by Chained Equations (MICE), outlier detection (OD): local outlier factor (LOF), classifier (CLS): multilayer perceptron (MLP))


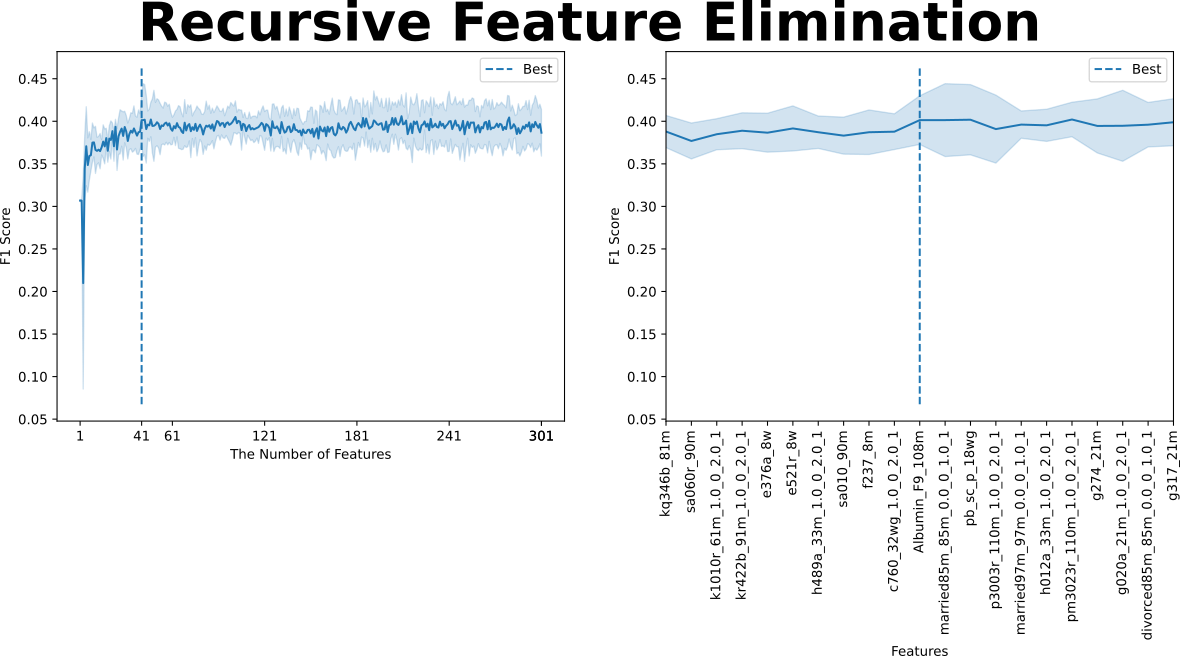


Supplementary Figure 15. Recursive Feature Elimination of 5-Fold Cross Validation of Dep18 for the best model pipeline (feature scaling (FS): robust, missing value imputation (MVI): Multivariate Imputation by Chained Equations (MICE), outlier detection (OD): local outlier factor (LOF), classifier (CLS): multilayer perceptron (MLP))


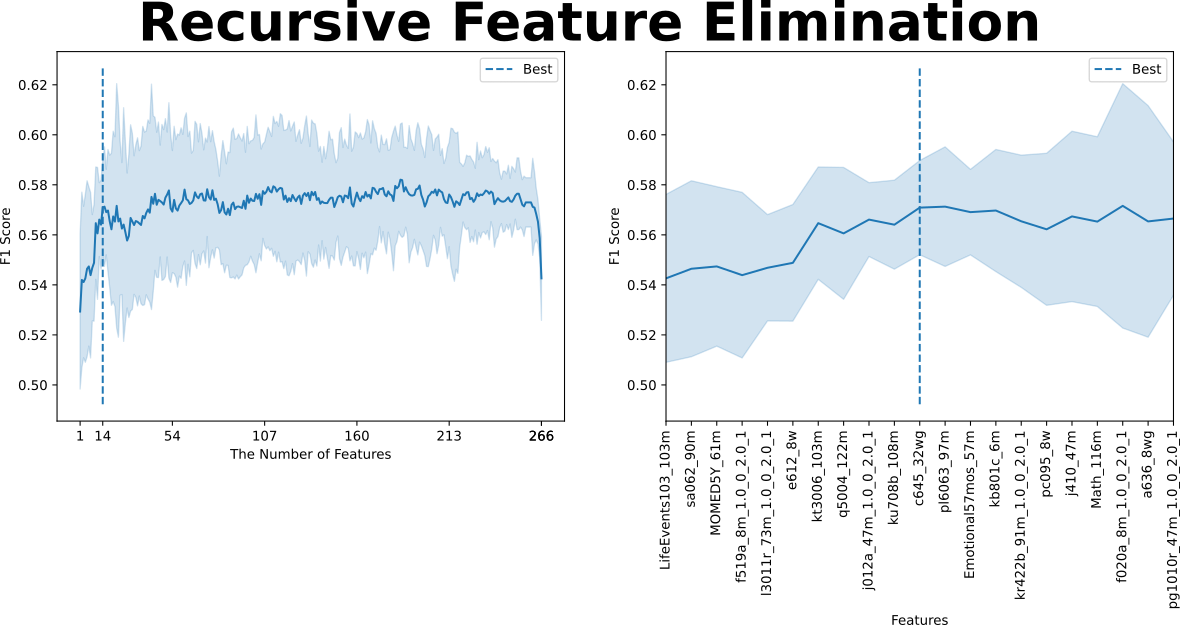


Supplementary Figure 16. Recursive Feature Elimination of 5-Fold Cross Validation of Dep12-18 for the best model pipeline (feature scaling (FS): minmax, missing value imputation (MVI): k-Nearest Neighbors (KNN), outlier detection (OD): None, classifier (CLS): AdaBoost)


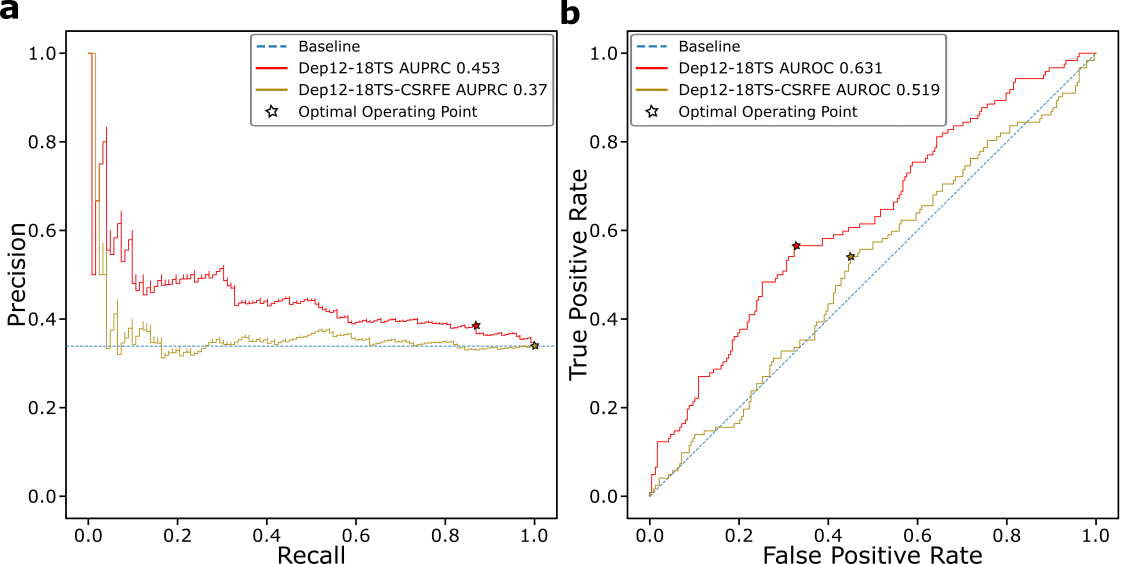


Supplementary Figure 17. Performance and evaluation of the time series machine learning models. a, b The precision-recall (PR) and receiver operating characteristic (ROC) curves of the best-performing time-series model for the Dep12-18TS dataset and the Dep12-18TS-CSRFE dataset obtained from the held-out test set. The Dep12-18TS-CSRFE dataset is the same as the Dep12-18TS dataset but only using features selected from the Dep12-18 recursive feature elimination (RFE). The operating point was selected using the F1-score.


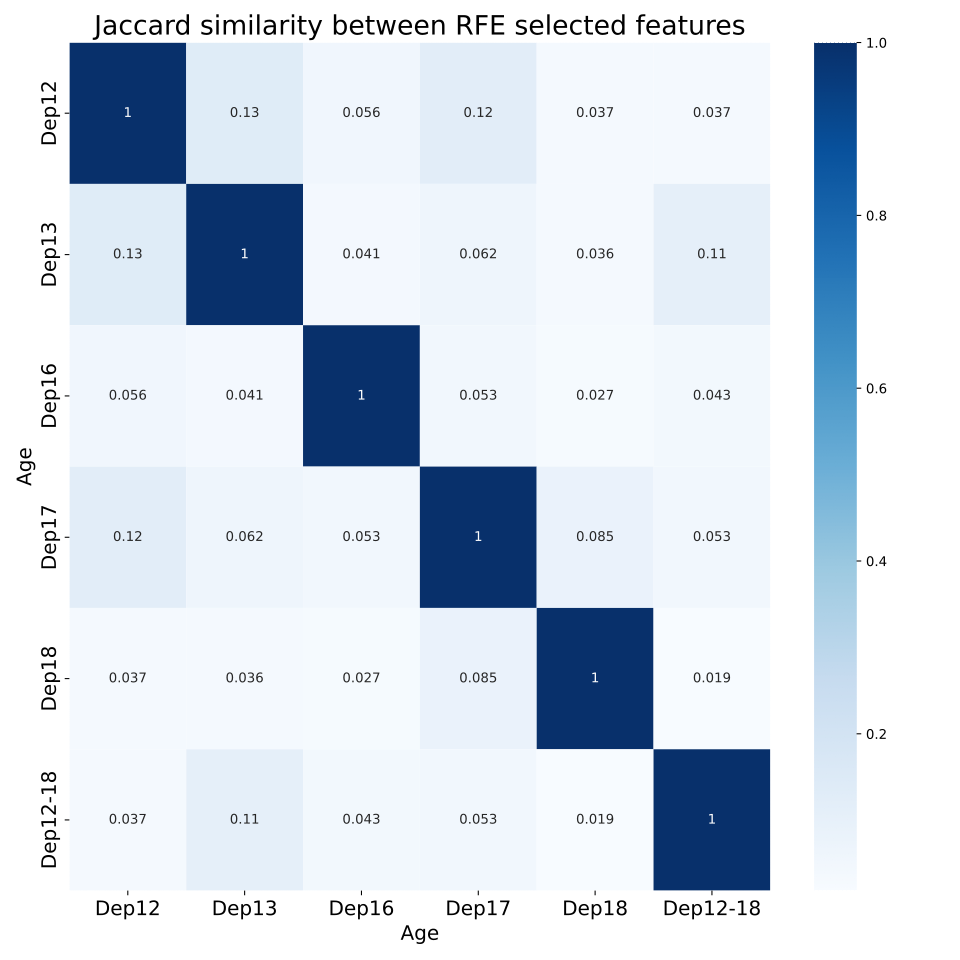


Supplementary Figure 18. Jaccard similarity index between recursive feature elimination (RFE) selected features.

#

# Supplementary Tables

Supplementary Table 1. Number of samples and features after each cross-sectional data cleaning step

| **Data Cleaning Step** | **Age 12 Data** | | **Age 13 Data** | | **Age 16 Data** | | **Age 17 Data** | | **Age 18 Data** | | **Age 12-18 Data** | |
| --- | --- | --- | --- | --- | --- | --- | --- | --- | --- | --- | --- | --- |
|  | **Samples** | **Features** | **Samples** | **Features** | **Samples** | **Features** | **Samples** | **Features** | **Samples** | **Features** | **Samples** | **Features** |
| Start with raw data | 15,645 | 6,163 | 15,645 | 6,163 | 15,645 | 6,163 | 15,645 | 6,163 | 15,645 | 6,163 | 15,645 | 6,163 |
| Merge features | 15,645 | 885 | 15,645 | 885 | 15,645 | 885 | 15,645 | 885 | 15,645 | 885 | 15,645 | 885 |
| Remove the samples with a missing target variable | 6,715 | 885 | 6,015 | 885 | 4,993 | 885 | 4,496 | 885 | 3,334 | 885 | 1,799 | 885 |
| Remove "ignored variables" (child ID) | 6,715 | 884 | 6,015 | 884 | 4,993 | 884 | 4,496 | 884 | 3,334 | 884 | 1,799 | 884 |
| Remove overly missing variables (>= 60% missing) | 6,715 | 763 | 6,015 | 763 | 4,993 | 765 | 4,496 | 767 | 3,334 | 767 | 1,799 | 767 |
| Remove constant variables | 6,715 | 763 | 6,015 | 762 | 4,993 | 764 | 4,496 | 765 | 3,334 | 764 | 1,799 | 762 |
| Remove variables >= age 11 | 6,715 | 754 | 6,015 | 753 | 4,993 | 755 | 4,496 | 754 | 3,334 | 753 | 1,799 | 751 |
| Remove features where the Pearson Correlation p-val is >= 0.05 | 6,715 | 255 | 6,015 | 279 | 4,993 | 338 | 4,496 | 318 | 3,334 | 299 | 1,799 | 266 |

Supplementary Table 2. Number of Features with Missing Value Ratios

| Missing Value Ratio Range | # Features | % Total | % Cumulative |
| --- | --- | --- | --- |
| [0, 0.1] | 2 | 0.2% | 0.2% |
| (0.1, 0.2] | 24 | 2.7% | 2.9% |
| (0.2, 0.3] | 102 | 11.5% | 14.5% |
| (0.3, 0.4] | 187 | 21.1% | 35.6% |
| (0.4, 0.5] | 227 | 25.6% | 61.2% |
| (0.5, 0.6] | 104 | 11.8% | 73.0% |
| (0.6, 0.7] | 90 | 10.2% | 83.2% |
| (0.7, 0.8] | 38 | 4.3% | 87.5% |
| (0.8, 0.9] | 10 | 1.1% | 88.6% |
| (0.9, 1) | 101 | 11.4% | 100% |
| Total | 885 | 100% | - |

**Supplementary Table 3. Grid Search Hyperparameter Space for Scikit-Learn(18)**

| **Model** | **Hyperparameters** | **Values** |
| --- | --- | --- |
| Decision Tree | Criterion Modes | gini, entropy |
|  | Splitter Modes | best, random |
|  | Max Depth | 1, 3, 5, …, 49 |
|  | Min Samples Split | 2, 4, 6, 8, 10 |
|  | Min Samples Leaf | 1, 3, 5, 7, 9 |
|  | Random State | 42 |
| Gaussian Naïve Bayes (NB) | None | None |
| Multinomial NB | None | None |
| Support Vector Classifier (SVC) | C | np.logspace(-3, -0.3, 10) |
|  | Max Iter | 100, 300, 500, 700, 900 |
|  | Random State | 42 |
| AdaBoost | Algorithm Modes | SAMME, SAMME.R |
|  | N_Estimators | 25, 50, 75, …, 500 |
|  | Learning Rate | 0.1, 0.3, 0.5, …, 1.9 |
|  | Random State | 42 |
| RandomForest | Criterion Modes | gini, entropy, logless |
|  | N_Estimators | 100, 200, 300, 400 |
|  | Max Depth | 1, 2, 3, 4 |
|  | Min Samples Split | 5, 10, 15, …, 50 |
|  | Max Features | sqrt, log2 |
|  | Class Weight | balanced, none |
|  | Random State | 42 |
| Multilayer Perceptron (MLP) | alpha | np.logspace(1.16, 2, 5) |
|  | Max Iter | 100, 300, 500, 700, 900 |
|  | Number of Hidden Layers | 1, 2 |
|  | Number of Neurons in Layer | 10, 30, 50, 70 |
|  | Random State | 42 |

**Supplementary Table 4. Best Hyperparameters Selected from Grid Search.** FS = Feature Scaling. MVI = Missing Value Imputation. OD = Outlier Detection. CLS = classifier. KNN = k-Nearest Neighbors. MICE = Multivariate Imputation by Chained Equations. MLP = multilayer perceptron.

| **Dataset** | **Pipeline** | **Hyperparameters** |
| --- | --- | --- |
| Dep12 | FS: robust, MVI: KNN, OD: LOF, CLS: AdaBoost | Algorithm Mode: SAMME.R |
|  |  | Learning Rate: 1.5 |
|  |  | N_Estimators: 25 |
|  |  | Random State: 42 |
| Dep13 | FS: robust, MVI: MICE, OD: None, CLS: MLP | alpha: 14.454397707459272 |
|  |  | Number of Hidden Layers: 1 |
|  |  | Number of Neurons in Layer: 10 |
|  |  | Max Iter: 100 |
|  |  | Random State: 42 |
| Dep16 | FS: minmax, MVI: KNN, OD: None, CLS: MLP | alpha: 14.454397707459272 |
|  |  | Number of Hidden Layers: 1 |
|  |  | Number of Neurons in Layer: 10 |
|  |  | Max Iter: 100 |
|  |  | Random State: 42 |
| Dep17 | FS: robust, MVI: MICE, OD: LOF, CLS: MLP | alpha: 38.018939632056124 |
|  |  | Number of Hidden Layers: 1 |
|  |  | Number of Neurons in Layer: 30 |
|  |  | Max Iter: 100 |
|  |  | Random State: 42 |
| Dep18 | FS: robust, MVI: MICE, OD: LOF, CLS: MLP | alpha: 23.442288153199215 |
|  |  | Number of Hidden Layers: 1 |
|  |  | Number of Neurons in Layer: 50 |
|  |  | Max Iter: 100 |
|  |  | Random State: 42 |
| Dep12-18 | FS: minmax, MVI: KNN, OD: None, CLS: AdaBoost | Algorithm Mode: SAMME |
|  |  | Learning Rate: 0.1 |
|  |  | N_Estimators: 25 |
|  |  | Random State: 42 |

Supplementary Table 5. Time Series Grid Search Hyperparameter Space. RNN = recurrent neural network. LSTM = long short-term memory.

| **Model** | **Hyperparameters** | **Values** |
| --- | --- | --- |
| RNN, LSTM | Number of Layers | 2, 4 |
|  | Hidden Layer Size | 100, 120, 140 |
|  | Dropout | 0.25, 0.5 |
|  | Batch Size | 16, 64 |
|  | Max Epochs | 50, 75, 100 |
|  | Learning Rate | 0.000001, 0.00001 |
|  | Random State | 42 |

Supplementary Table 6. Best Hyperparameters Selected from Time Series Grid Search. FS = Feature Scaling. MVI = Missing Value Imputation. OD = Outlier Detection. CLS = classifier. NOCB = Next Observation Carried Backwards. RNN = recurrent neural network, LSTM = long short-term memory.

| Dep12-18TS | FS: standard, MVI: NOCB, OD: None, CLS: RNN | Number of Layers: 4 |
| --- | --- | --- |
|  |  | Hidden Layer Size: 120 |
|  |  | Dropout: 0.5 |
|  |  | Batch Size: 64 |
|  |  | Max Epochs: 50 |
|  |  | Learning Rate: 0.00001 |
|  |  | Random State: 42 |
| Dep12-18TS-CSRFE | FS: standard, MVI: Simple, OD: None, CLS: LSTM | Number of Layers: 2 |
|  |  | Hidden Layer Size: 120 |
|  |  | Dropout: 0.5 |
|  |  | Batch Size: 64 |
|  |  | Max Epochs: 100 |
|  |  | Learning Rate: 0.00001 |
|  |  | Random State: 42 |

Supplementary Table 7. Result of running model selection pipeline on the 6 datasets. The following model combination of feature scaling (FS), missing value imputation (MVI), outlier detection (OD), and binary classification (CLS) were selected for each dataset. Note that model selection is based off the validation set performance, so this F1 score is the validation set’s F1 score. KNN = k-Nearest Neighbors. MICE = Multivariate Imputation by Chained Equations. LOF = Local Outlier Factor. MLP = Multilayer Perceptron.

| **Data** | **FS** | **MVI** | **OD** | **CLS** | **F1 Score** |
| --- | --- | --- | --- | --- | --- |
| **Dep12** | Robust | KNN | LOF | AdaBoost | 0.215 |
| **Dep13** | Robust | MICE | None | MLP | 0.268 |
| **Dep16** | MinMax | KNN | None | MLP | 0.358 |
| **Dep17** | Robust | MICE | LOF | MLP | 0.380 |
| **Dep18** | Robust | MICE | LOF | MLP | 0.387 |
| **Dep12-18** | MinMax | KNN | None | AdaBoost | 0.543 |

Supplementary Table 8. Confusion matrix information for the Dep12 dataset. Baseline = Baseline confusion matrix where a classifier only predicts one class ‘Depressed’ (label 1). Before recursive feature elimination (RFE) = Confusion matrix for the best model pipeline (FS: robust, MVI: KNN, OD: LOF, CLS: AdaBoost). After RFE = Confusion matrix for the best model pipeline using only the features selected from the RFE. TP = True Positive (where positive = depressed), TN = True Negative, FP = False Positive, FN = False Negative, PP = Predicted Positive, PN = Predicted Negative, Pos = total number of samples that are positive, Neg = total number of samples that are negative, Prec = Precision, Spec = Specificity NPV = Negative Predicted Value, Acc = Accuracy F1 = F1 Score

| **Model** | **TP** | **TN** | **FP** | **FN** | **PP** | **PN** | **Pos** | **Neg** | **Prec** | **NPV** | **Recall** | **Spec** | **Acc** | **F1** |
| --- | --- | --- | --- | --- | --- | --- | --- | --- | --- | --- | --- | --- | --- | --- |
| Baseline | 62 | 0 | 1,214 | 0 | 1,276 | 0 | 62 | 1,214 | 0.049 | N/A | 1.0 | 0.0 | 0.049 | 0.093 |
| Before RFE | 16 | 1,107 | 107 | 46 | 123 | 1,153 | 62 | 1,214 | 0.13 | 0.96 | 0.258 | 0.912 | 0.88 | 0.173 |
| After RFE | 12 | 1,109 | 105 | 50 | 117 | 1,159 | 62 | 1,214 | 0.103 | 0.957 | 0.194 | 0.914 | 0.879 | 0.134 |

**Supplementary Table 9. Confusion matrix information for the Dep13 dataset.** Baseline = Baseline confusion matrix where a classifier only predicts one class ‘Depressed’ (label 1). Before recursive feature elimination (RFE) = Confusion matrix for the best model pipeline (FS: robust, MVI: MICE, OD: None, CLS: MLP). After RFE = Confusion matrix for the best model pipeline using only the features selected from the RFE. TP = True Positive (where positive = depressed), TN = True Negative, FP = False Positive, FN = False Negative, PP = Predicted Positive, PN = Predicted Negative, Pos = total number of samples that are positive, Neg = total number of samples that are negative, Prec = Precision, Spec = Specificity NPV = Negative Predicted Value, Acc = Accuracy F1 = F1 Score

| **Model** | **TP** | **TN** | **FP** | **FN** | **PP** | **PN** | **Pos** | **Neg** | **Prec** | **NPV** | **Recall** | **Spec** | **Acc** | **F1** |
| --- | --- | --- | --- | --- | --- | --- | --- | --- | --- | --- | --- | --- | --- | --- |
| Baseline | 102 | 0 | 1,101 | 0 | 1,203 | 0 | 102 | 1,101 | 0.085 | N/A | 1.0 | 0.0 | 0.085 | 0.156 |
| Before RFE | 51 | 785 | 316 | 51 | 367 | 836 | 102 | 1,101 | 0.139 | 0.939 | 0.5 | 0.713 | 0.695 | 0.217 |
| After RFE | 60 | 775 | 326 | 42 | 386 | 817 | 102 | 1,101 | 0.155 | 0.949 | 0.588 | 0.704 | 0.694 | 0.246 |

**Supplementary Table 10. Confusion matrix information for the Dep16 dataset.** Baseline = Baseline confusion matrix where a classifier only predicts one class ‘Depressed’ (label 1). Before recursive feature elimination (RFE) = Confusion matrix for the best model pipeline (FS: minmax, MVI: KNN, OD: None, CLS: MLP). After RFE = Confusion matrix for the best model pipeline using only the features selected from the RFE. TP = True Positive (where positive = depressed), TN = True Negative, FP = False Positive, FN = False Negative, PP = Predicted Positive, PN = Predicted Negative, Pos = total number of samples that are positive, Neg = total number of samples that are negative, Prec = Precision, Spec = Specificity NPV = Negative Predicted Value, Acc = Accuracy F1 = F1 Score

| **Model** | **TP** | **TN** | **FP** | **FN** | **PP** | **PN** | **Pos** | **Neg** | **Prec** | **NPV** | **Recall** | **Spec** | **Acc** | **F1** |
| --- | --- | --- | --- | --- | --- | --- | --- | --- | --- | --- | --- | --- | --- | --- |
| Baseline | 156 | 0 | 843 | 0 | 999 | 0 | 156 | 843 | 0.156 | N/A | 1.0 | 0.0 | 0.156 | 0.27 |
| Before RFE | 93 | 505 | 338 | 63 | 431 | 568 | 156 | 843 | 0.216 | 0.889 | 0.596 | 0.599 | 0.599 | 0.317 |
| After RFE | 117 | 381 | 462 | 39 | 579 | 420 | 156 | 843 | 0.202 | 0.907 | 0.75 | 0.452 | 0.498 | 0.318 |

Supplementary Table 11. Confusion matrix information for the Dep17 dataset. Baseline = Baseline confusion matrix where a classifier only predicts one class ‘Depressed’ (label 1). Before recursive feature elimination (RFE) = Confusion matrix for the best model pipeline (FS: robust, MVI: MICE, OD: LOF, CLS: MLP). After RFE = Confusion matrix for the best model pipeline using only the features selected from the RFE. TP = True Positive (where positive = depressed), TN = True Negative, FP = False Positive, FN = False Negative, PP = Predicted Positive, PN = Predicted Negative, Pos = total number of samples that are positive, Neg = total number of samples that are negative, Prec = Precision, Spec = Specificity NPV = Negative Predicted Value, Acc = Accuracy F1 = F1 Score

| **Model** | **TP** | **TN** | **FP** | **FN** | **PP** | **PN** | **Pos** | **Neg** | **Prec** | **NPV** | **Recall** | **Spec** | **Acc** | **F1** |
| --- | --- | --- | --- | --- | --- | --- | --- | --- | --- | --- | --- | --- | --- | --- |
| Baseline | 151 | 0 | 704 | 0 | 855 | 0 | 151 | 704 | 0.177 | N/A | 1.0 | 0.0 | 0.177 | 0.3 |
| Before RFE | 89 | 405 | 299 | 62 | 388 | 467 | 151 | 704 | 0.229 | 0.867 | 0.589 | 0.575 | 0.578 | 0.33 |
| After RFE | 83 | 453 | 251 | 68 | 334 | 521 | 151 | 704 | 0.249 | 0.869 | 0.55 | 0.643 | 0.627 | 0.342 |

Supplementary Table 12. Confusion matrix information for the Dep18 dataset. Baseline = Baseline confusion matrix where a classifier only predicts one class ‘Depressed’ (label 1). Before recursive feature elimination (RFE) = Confusion matrix for the best model pipeline (FS: robust, MVI: MICE, OD: LOF, CLS: MLP). After RFE = Confusion matrix for the best model pipeline using only the features selected from the RFE. TP = True Positive (where positive = depressed), TN = True Negative, FP = False Positive, FN = False Negative, PP = Predicted Positive, PN = Predicted Negative, Pos = total number of samples that are positive, Neg = total number of samples that are negative, Prec = Precision, Spec = Specificity NPV = Negative Predicted Value, Acc = Accuracy F1 = F1 Score

| **Model** | **TP** | **TN** | **FP** | **FN** | **PP** | **PN** | **Pos** | **Neg** | **Prec** | **NPV** | **Recall** | **Spec** | **Acc** | **F1** |
| --- | --- | --- | --- | --- | --- | --- | --- | --- | --- | --- | --- | --- | --- | --- |
| Baseline | 119 | 0 | 515 | 0 | 634 | 0 | 119 | 515 | 0.188 | N/A | 1.0 | 0.0 | 0.188 | 0.316 |
| Before RFE | 61 | 381 | 134 | 58 | 195 | 439 | 119 | 515 | 0.313 | 0.868 | 0.513 | 0.74 | 0.697 | 0.389 |
| After RFE | 75 | 300 | 215 | 44 | 290 | 344 | 119 | 515 | 0.259 | 0.872 | 0.63 | 0.583 | 0.591 | 0.367 |

Supplementary Table 13. Confusion matrix information for the Dep12-18 dataset. Baseline = Baseline confusion matrix where a classifier only predicts one class ‘Depressed’ (label 1). Before recursive feature elimination (RFE) = Confusion matrix for the best model pipeline (FS: minmax, MVI: KNN, OD: None, CLS: AdaBoost). After RFE = Confusion matrix for the best model pipeline using only the features selected from the RFE. TP = True Positive (where positive = depressed), TN = True Negative, FP = False Positive, FN = False Negative, PP = Predicted Positive, PN = Predicted Negative, Pos = total number of samples that are positive, Neg = total number of samples that are negative, Prec = Precision, Spec = Specificity NPV = Negative Predicted Value, Acc = Accuracy F1 = F1 Score

| **Model** | **TP** | **TN** | **FP** | **FN** | **PP** | **PN** | **Pos** | **Neg** | **Prec** | **NPV** | **Recall** | **Spec** | **Acc** | **F1** |
| --- | --- | --- | --- | --- | --- | --- | --- | --- | --- | --- | --- | --- | --- | --- |
| Baseline | 125 | 0 | 235 | 0 | 360 | 0 | 125 | 235 | 0.347 | N/A | 1.0 | 0.0 | 0.347 | 0.515 |
| Before RFE | 91 | 99 | 136 | 34 | 227 | 133 | 125 | 235 | 0.401 | 0.744 | 0.728 | 0.421 | 0.528 | 0.517 |
| After RFE | 100 | 88 | 147 | 25 | 247 | 113 | 125 | 235 | 0.405 | 0.779 | 0.8 | 0.374 | 0.522 | 0.538 |

Supplementary Table 14. Confusion matrix information for the Dep12-18TS and Dep12-18TS-CSRFE dataset. Baseline = Baseline confusion matrix where a classifier only predicts one class ‘Depressed’ (label 1). Before recursive feature elimination (RFE) = Confusion matrix for the best model pipeline for Dep12-18TS (FS: standard, MVI: NOCB, OD: None, CLS: RNN). After RFE = Confusion matrix for the best model pipeline for Dep12-18TS-CSRFE (FS: standard, MVI: Simple, OD: None, CLS: LSTM). TP = True Positive (where positive = depressed), TN = True Negative, FP = False Positive, FN = False Negative, PP = Predicted Positive, PN = Predicted Negative, Pos = total number of samples that are positive, Neg = total number of samples that are negative, Prec = Precision, Spec = Specificity NPV = Negative Predicted Value, Acc = Accuracy F1 = F1 Score

| **Model** | **TP** | **TN** | **FP** | **FN** | **PP** | **PN** | **Pos** | **Neg** | **Prec** | **NPV** | **Recall** | **Spec** | **Acc** | **F1** |
| --- | --- | --- | --- | --- | --- | --- | --- | --- | --- | --- | --- | --- | --- | --- |
| Baseline | 122 | 0 | 238 | 0 | 360 | 0 | 122 | 238 | 0.339 | N/A | 1.0 | 0.0 | 0.339 | 0.506 |
| Before RFE | 77 | 120 | 118 | 45 | 195 | 165 | 122 | 238 | 0.395 | 0.727 | 0.631 | 0.504 | 0.547 | 0.486 |
| After RFE | 65 | 130 | 108 | 57 | 173 | 187 | 122 | 238 | 0.376 | 0.695 | 0.533 | 0.546 | 0.542 | 0.441 |

Supplementary Table 15. Top 20 features identified after performing the recursive feature elimination (RFE). Within each dataset, we first ranked the RFE selected features according to the RFE results. We then sorted the features according to their number of appearances across the 6 datasets (e.g., # of appearance = 6 means that this feature was selected by RFE for all 6 datasets). The features that share the same # of appearances were further sorted incrementally by their average rank. We also included the Pearson correlation and Pearson correlation ranks from these features in the training data for all 6 datasets.

| **Feature** | **# of RFE appearance** | **Avg. RFE Rank** | **RFE Rank Dep12** | **RFE Rank Dep13** | **RFE Rank Dep16** | **RFE Rank Dep17** | **RFE Rank Dep18** | **RFE Rank Dep12-18** | **Avg. Pearson Rank** | **Pearson Rank Dep12** | **Pearson Rank Dep13** | **Pearson Rank Dep16** | **Pearson Rank Dep17** | **Pearson Rank Dep18** | **Pearson Rank Dep12-18** | **Pearson Correlation Dep12** | **Pearson Correlation Dep13** | **Pearson Correlation Dep16** | **Pearson Correlation Dep17** | **Pearson Correlation Dep18** | **Pearson Correlation Dep12-18** |
| --- | --- | --- | --- | --- | --- | --- | --- | --- | --- | --- | --- | --- | --- | --- | --- | --- | --- | --- | --- | --- | --- |
| Child sex (1=male, 2=female) @ 0m | 6 | 6.5 | 3 | 2 | 2 | 30 | 1 | 1 | 16.8 | 79 | 3 | 3 | 12 | 1 | 3 | 0.06 | 0.13 | 0.15 | 0.1 | 0.15 | 0.19 |
| Depression Score at Focus 10 assessment: F10+ (Higher = more) @ 120m | 5 | 8 | 4 | 1 | 30 | 3 | - | 2 | 4.3 | 1.5 | 2 | 1.5 | 2 | 17 | 1.5 | 0.19 | 0.15 | 0.17 | 0.15 | 0.12 | 0.22 |
| SDQI - Child is pleased with physical appearance (Higher = more good looking.) @ 116m | 3 | 24.3 | - | 11 | - | 58 | 4 | - | 58.3 | 13 | 9 | 54 | 145 | 65 | 64 | -0.09 | -0.09 | -0.08 | -0.06 | -0.09 | -0.11 |
| DV: SDQ Emotional Symptoms Score (prorated) (Higher = more.) @ 81m |  | 28.3 | 28 | 16 | - | 41 | - | - | 108.8 | 115 | 200 | 22 | 112 | 79 | 125 | 0.05 | 0.04 | 0.1 | 0.07 | 0.08 | 0.08 |
| SMFQ depression score >=12 yes/no at age 10 (Higher=more) @ 127m |  | 34.7 | 1 | - | - | 82 | 21 | - | 55.2 | 3 | 10 | 38 | 24 | 216 | 40 | 0.18 | 0.09 | 0.09 | 0.1 | 0.05 | 0.12 |
| DV: Particular fears score (prorated) (Higher = more) @ 91m |  | 36 | 37 | 9 | - | 62 | - | - | 104.2 | 144 | 19 | 95 | 202 | 36 | 129 | 0.04 | 0.07 | 0.07 | 0.05 | 0.1 | 0.08 |
| Freq mum felt depressed in past month (Range 1-3, higher = more) @ 33m | 2 | 3.5 | 5 | - | - | 2 | - | - | 88.3 | 33 | 90 | 62 | 78 | 171 | 96 | -0.08 | -0.05 | -0.08 | -0.08 | -0.06 | -0.09 |
| Weighted Life Events Score (Higher=more) @ 103m |  | 4 | - | 4 | - | - | - | 4 | 126.2 | 18 | 14 | 286 | 200 | 177 | 62 | 0.08 | 0.08 | 0.04 | 0.05 | 0.06 | 0.11 |
| Mother's educational qualification 5 years (Range 0-5, higher = better) @ 61m |  | 8.5 | - | - | - | 11 | - | 6 | 139.8 | - | - | 102 | 61 | 136 | 260 | - | - | -0.07 | -0.08 | -0.07 | -0.04 |
| B15: Child is influenced by friends or mates (1= Very strongly, 5 = Never) @ 108m |  | 11.5 | - | - | - | 17 | 6 | - | 192.2 | 224 | 263 | 254 | 86 | 163 | 163 | -0.03 | -0.02 | -0.04 | -0.08 | -0.06 | -0.07 |
| DV: SDQ Peer Problems Score (prorated) (Higher = more.) @ 81m |  | 14 | - | 3 | - | 25 | - | - | 91.3 | 30 | 5 | 112 | 59 | 255 | 87 | 0.08 | 0.11 | 0.06 | 0.08 | 0.04 | 0.1 |
| SDQI - global self-esteem (Higher = better at school work) @ 116m |  | 14.5 | 22 | 7 | - | - | - | - | 25.2 | 4 | 6 | 6 | 49 | 11 | 75 | -0.11 | -0.1 | -0.12 | -0.08 | -0.12 | -0.1 |
| EAS emotionality (prorated) (Higher = more) @ 69m |  | 16 | 18 | 14 | - | - | - | - | 72.5 | 44 | 45 | 76 | 130 | 32 | 108 | 0.07 | 0.06 | 0.07 | 0.06 | 0.11 | 0.09 |
| B26: Mother got married since the study child's 6th birthday (no=0, yes = 1) @ 110m |  | 16 | - | - | - | 16 | 16 | - | 191.3 | - | - | - | 319 | 107 | 148 | - | - | - | 0.02 | 0.07 | 0.08 |
| Child not wanted by partner (1=yes, 2 = no) @ 8m |  | 17.5 | - | - | - | 9 | 26 | - | 250.5 | - | - | 308 | 154 | 277 | 263 | - | - | -0.03 | -0.06 | -0.04 | -0.04 |
| DV: SDQ Hyperactivity Score (prorated) (Higher = more.) @ 81m |  | 17.5 | - | - | 4 | - | 31 | - | 169.3 | 97 | 179 | 265 | 97 | 279 | 99 | 0.05 | 0.04 | 0.04 | 0.07 | 0.04 | 0.09 |
| DV: SDQ - Peer problems score (prorated) (Higher = more.) @ 108m |  | 19 | 19 | - | - | 19 | - | - | 34.7 | 5 | 8 | 75 | 44 | 51 | 25 | 0.1 | 0.09 | 0.07 | 0.09 | 0.1 | 0.13 |
| Social support score: modes/missing data (0-30, higher more support) @ 21wg |  | 19 | 30 | 8 | - | - | - | - | 110.2 | 168 | 83 | 36 | 25 | 105 | 244 | -0.04 | -0.05 | -0.09 | -0.1 | -0.07 | -0.05 |
| F&S Friends score: F@8 (Lower = more positive friendships) @ 96m |  | 21 | - | - | 21 | 21 | - | - | 204 | 136 | 87 | 311 | 232 | - | 254 | 0.04 | 0.05 | 0.03 | 0.05 | - | 0.05 |
| Child score on moods and feelings questionnaire (depression) at 10 years (Higher=more) @ 127m |  | 23.5 | - | - | 32 | 15 | - | - | 3.8 | 1.5 | 1 | 1.5 | 1 | 16 | 1.5 | 0.19 | 0.15 | 0.17 | 0.15 | 0.12 | 0.22 |

**Supplementary Table 16. Leading five predictors of depression outcomes at each age from recursive feature elimination (RFE)**. Columns show the rank of each predictor based on RFE, such that 1 = the first-ranked predictor. Abbreviations: m = month; SMFQ = Short Mood and Feelings Questionnaire; SDQ = Strengths and Difficulties Questionnaire.

| Predictive feature | Age 12 | Age 13 | Age 16 | Age 17 | Age 18 | Age 12-18 |
| --- | --- | --- | --- | --- | --- | --- |
| Female sex | 3 | 2 | 2 |  | 1 | 1 |
| Child Depression Score at age 10 | 4 | 1 |  | 3 |  | 2 |
| Mom's opinion of neighborhood 122m |  |  |  |  |  | 3 |
| Child Life Events Score at 103m |  | 4 |  |  |  | 4 |
| Frequency child does required homework 90m |  |  |  |  |  | 5 |
| Self Esteem: Scholastic Competence Score Age 8 |  |  | 1 |  |  |  |
| SMFQ depression score age 10 | 1 |  |  |  |  |  |
| Mom married at 8 weeks gestation |  |  |  | 1 |  |  |
| Child's relationship with parents 116m | 2 |  |  |  |  |  |
| Teacher's rating of child for age - general knowledge 90m | |  |  |  | 2 |  |
| Mother Edinburgh Postnatal Depression Score 21m |  |  |  |  | 3 |  |
| SDQ Peer Problems Score 81m |  | 3 |  |  |  |  |
| Mother took depression pills 47m |  |  | 3 |  |  |  |
| Freq mom felt depressed in past month 33m | 5 |  |  | 2 |  |  |
| SDQ Hyperactivity Score 81m |  |  | 4 |  |  |  |
| Mom Edinburgh Postnatal Depression Score 33m |  |  |  | 4 |  |  |
| Child is pleased with physical appearance 116m |  |  |  |  | 4 |  |
| Mom has ever had severe depression 97m |  |  |  | 5 |  |  |
| Mom's partner social support score 61m |  |  |  |  | 5 |  |
| Child is good at athletics 116m |  | 5 |  |  |  |  |
| Mom took depression pills 33m |  |  | 5 |  |  |  |

# References

1. Webber W, Moffat A, Zobel J. A similarity measure for indefinite rankings. ACM Transactions on Information Systems (TOIS). 2010;28(4):1–38.

2. Kendall MG. A new measure of rank correlation. Biometrika. 1938 Jun 1;30(1–2):81–93.

3. Tate AE, McCabe RC, Larsson H, Lundström S, Lichtenstein P, Kuja-Halkola R. Predicting mental health problems in adolescence using machine learning techniques. PloS one. 2020;15(4):e0230389.

4. Salganik MJ, Lundberg I, Kindel AT, Ahearn CE, Al-Ghoneim K, Almaatouq A, et al. Measuring the predictability of life outcomes with a scientific mass collaboration. Proceedings of the National Academy of Sciences. 2020;117(15):8398–403.

5. Vijayakumar N, de Macks ZO, Shirtcliff EA, Pfeifer JH. Puberty and the human brain: Insights into adolescent development. Neuroscience & Biobehavioral Reviews. 2018;92:417–36.

6. Alley RB, Emanuel KA, Zhang F. Advances in weather prediction. Science. 2019;363(6425):342–4.

7. Franklin JC. Psychological primitives can make sense of biopsychosocial factor complexity in psychopathology. BMC medicine. 2019;17(1):1–8.

8. Hammen C. Depression and stressful environments: Identifying gaps in conceptualization and measurement. Anxiety, Stress, & Coping. 2016;29(4):335–51.

9. Cellini P, Pigoni A, Delvecchio G, Moltrasio C, Brambilla P. Machine learning in the prediction of postpartum depression: A review. Journal of Affective Disorders. 2022;

10. Chahal R, Gotlib IH, Guyer AE. Research Review: Brain network connectivity and the heterogeneity of depression in adolescence--a precision mental health perspective. Journal of Child Psychology and Psychiatry. 2020;61(12):1282–98.

11. Schmaal L, Hibar DP, Sämann PG, Hall GB, Baune BT, Jahanshad N, et al. Cortical abnormalities in adults and adolescents with major depression based on brain scans from 20 cohorts worldwide in the ENIGMA Major Depressive Disorder Working Group. Molecular psychiatry. 2017;22(6):900–9.

12. Schmaal L, Veltman DJ, van Erp TGM, Sämann PG, Frodl T, Jahanshad N, et al. Subcortical brain alterations in major depressive disorder: findings from the ENIGMA Major Depressive Disorder working group. Molecular psychiatry. 2016;21(6):806–12.

13. De Asis-Cruz J, Andescavage N, Limperopoulos C. Adverse Prenatal Exposures and Fetal Brain Development: Insights from Advanced Fetal MRI. Biological Psychiatry: Cognitive Neuroscience and Neuroimaging. 2021;

14. Xiang Q, Chen K, Peng L, Luo J, Jiang J, Chen Y, et al. Prediction of the trajectories of depressive symptoms among children in the adolescent brain cognitive development (ABCD) study using machine learning approach. Journal of Affective Disorders. 2022;310:162–71.

15. Foland-Ross LC, Sacchet MD, Prasad G, Gilbert B, Thompson PM, Gotlib IH. Cortical thickness predicts the first onset of major depression in adolescence. International Journal of Developmental Neuroscience. 2015;46:125–31.

16. Cai N, Revez JA, Adams MJ, Andlauer TFM, Breen G, Byrne EM, et al. Minimal phenotyping yields genome-wide association signals of low specificity for major depression. Nature Genetics. 2020;52(4):437–47.

17. Chai X, Gu H, Li F, Duan H, Hu X, Lin K. Deep learning for irregularly and regularly missing data reconstruction. Sci Rep. 2020 Feb 24;10(1):3302.

18. Pedregosa F, Varoquaux G, Gramfort A, Michel V, Thirion B, Grisel O, et al. Scikit-learn: Machine Learning in Python. Journal of Machine Learning Research. 2011;12:2825–30.
